# Spatial Clustering of Porcine Cysticercosis in Mbulu District, Northern Tanzania

**DOI:** 10.1371/journal.pntd.0000652

**Published:** 2010-04-06

**Authors:** Helena A. Ngowi, Ayub A. Kassuku, Hélène Carabin, James E. D. Mlangwa, Malongo R. S. Mlozi, Boniface P. Mbilinyi, Arve L. Willingham

**Affiliations:** 1 Department of Veterinary Medicine and Public Health, Sokoine University of Agriculture, Morogoro, Tanzania; 2 Department of Veterinary Microbiology and Parasitology, Sokoine University of Agriculture, Morogoro, Tanzania; 3 Department of Biostatistics and Epidemiology, College of Public Health, University of Oklahoma Health Sciences Center, Oklahoma City, Oklahoma, United States of America; 4 Department of Agricultural Education and Extension, Sokoine University of Agriculture, Morogoro, Tanzania; 5 Department of Agricultural Engineering and Land Planning, Sokoine University of Agriculture, Morogoro, Tanzania; 6 WHO/FAO Collaborating Center for Research and Training on Emerging and Other Parasitic Zoonoses, Danish Centre for Experimental Parasitology, The Faculty of Life Sciences, University of Copenhagen, Frederiksberg, Denmark; Universidad Peruana Cayetano Heredia, Peru

## Abstract

**Background:**

Porcine cysticercosis is caused by a zoonotic tapeworm, *Taenia solium*, which causes serious disease syndromes in human. Effective control of the parasite requires knowledge on the burden and pattern of the infections in order to properly direct limited resources. The objective of this study was to establish the spatial distribution of porcine cysticercosis in Mbulu district, northern Tanzania, to guide control strategies.

**Methodology/Principal Findings:**

This study is a secondary analysis of data collected during the baseline and follow-up periods of a randomized community trial aiming at reducing the incidence rate of porcine cysticercosis through an educational program. At baseline, 784 randomly selected pig-keeping households located in 42 villages in 14 wards were included. Lingual examination of indigenous pigs aged 2–12 (median 8) months, one randomly selected from each household, were conducted. Data from the control group of the randomized trial that included 21 of the 42 villages were used for the incidence study. A total of 295 pig-keeping households were provided with sentinel pigs (one each) and reassessed for cysticercosis incidence once or twice for 2–9 (median 4) months using lingual examination and antigen ELISA. Prevalence of porcine cysticercosis was computed in Epi Info 3.5. The prevalence and incidence of porcine cysticercosis were mapped at household level using ArcView 3.2. K functions were computed in R software to assess general clustering of porcine cysticercosis. Spatial scan statistics were computed in SatScan to identify local clusters of the infection. The overall prevalence of porcine cysticercosis was 7.3% (95% CI: 5.6, 9.4; n = 784). The K functions revealed a significant overall clustering of porcine cysticercosis incidence for all distances between 600 m and 5 km from a randomly chosen case household based on Ag-ELISA. Lingual examination revealed clustering from 650 m to 6 km and between 7.5 and 10 km. The prevalence study did not reveal any significant clustering by this method. Spatial scan statistics found one significant cluster of porcine cysticercosis prevalence (P = 0.0036; n = 370). In addition, the analysis found one large cluster of porcine cysticercosis incidence based on Ag-ELISA (P = 0.0010; n = 236) and two relatively small clusters of incidence based on lingual examination (P = 0.0012 and P = 0.0026; n = 241). These clusters had similar spatial location and included six wards, four of which were identified as high risk areas of porcine cysticercosis.

**Conclusion/Significance:**

This study has identified local clusters of porcine cysticercosis in Mbulu district, northern Tanzania, where limited resources for control of *T. solium* could be directed. Further studies are needed to establish causes of clustering to institute appropriate interventions.

## Author Summary

*Taenia solium* is a tapeworm that causes two different disease conditions. In its adult stage, it inhabits the small intestine of human, a condition known as taeniosis, which is characterised by mild symptoms including abdominal disconfort. In the larval stage, *T. solium* can infect humans and various animal species, mainly pigs, causing cysticercosis. Taeniosis is acquired through consumption of inadequately cooked infected meat, while cysticercosis is acquired through ingestion of tapeworm eggs in foodstuffs contaminated with faeces from a human tapeworm carrier. Cysticercosis of human central nervous tissues (neurocysticercosis) causes serious syndromes such as epilepsy. Transmission of *T. solium* is facilitated by several factors such as presence of tapeworm carriers, poor sanitation and poor pig husbandry, which allow pigs to access human faeces. Nevertheless, the role of these factors in parasite transmission may vary with different cultural settings. Following an incidence and a prevalence studies in a rural area of northern Tanzania, there was a significant spatial clustering of porcine cysticerocis, suggesting focal distribution of transmission risk factors, which could be targeted for interventions. The study also revealed that despite the low sensitivity of the lingual examination method to detect porcine cysticercosis, it could highlight the potential ‘hotspots’ of the infection.

## Introduction

Porcine cysticercosis is caused by the larval stage of the tapeworm *Taenia solium*, which also infects human and may cause serious neurological disorders such as epilepsy [1]. The lifecycle of *T. solium* involves a human as the sole natural definitive host carrying the adult parasite in the small intestine resulting from consumption of insufficiently cooked meat infested with parasite larvae. A person infested with the adult parasite can transmit the infection to intermediate hosts including pigs and humans through faecal-oral transmission such as faecal contamination of foodstuffs or by other means leading to cysticercosis. Human neurocysticercosis, the infection of the central nervous system by the larval stages of *T. solium*, has been found to account for up to 50% of epilepsy in areas where *T. solium* is endemic [1].

Eradication of *T. solium* is considered epidemiologically possible because of several factors, including the fact that the human being is the only source of infection to the intermediate hosts, commonly pigs, and that the latter can be used to monitor the dynamics of the infection because of their short life span [2]. In addition, efficacious diagnostic and therapeutic agents for the parasite are currently available, which facilitate surveillance and control activities. Because of this perceived simplicity, the International Taskforce for Disease Eradication recommended that elimination of *T. solium* be implemented in a sizeable geographical area [3]. Nevertheless, *T. solium* has not been eliminated in developing countries because of poverty combined with a lack of sanitation, the need to involve both the agricultural and health sectors in control efforts, and the lack of political will, which may be attributed to poor knowledge on the presence, magnitude and impact of the parasite. Because of limited resources in many developing countries, control efforts for *T. solium* should be targeted to specific (preferably small) areas of high endemicity to reduce the burden, which could possibly eliminate the parasite in the long run. However, the spatial patterns of *T. solium* infections in many endemic settings are not well known. Such information could be used to guide efficient and effective utilization of limited financial and personnel resources through targeting interventions to areas of high endemicity. Analysis of spatial point pattern of porcine cysticercosis in an endemic situation would be very valuable in this aspect.

Spatial pattern analysis has been found to be useful in the control of other human helminth infections elsewhere. It may be a key factor in understanding disease transmission when there is strong correlation between the spatial distribution of the disease and hosts or transmission risk factors. For example, a study in coastal Kenya found a significant clustering of urinary schistosomiasis around a water-contact site with high numbers of snails shedding *Schistosoma haematobium* cercariae [4]. Similar findings have been reported in Uganda [5]. A study in Belgium and The Netherlands established a spatial correlation of the fox tapeworm *Echinococcus multilocularis* in Red foxes [6]. Nevertheless, this is the first time that spatial pattern analysis is being applied in *T. solium* research in Africa.

A study in Peru observed significant clustering of human cysticercosis seropositivity by the household whether or not the household had a tapeworm carrier [7]. Widdowson and others reported household clustering of porcine cysticercosis in Mexico [8]. However, both of these studies did not disentangle the clustering of the infections that could occur just because of natural environmental variation in pig or human population density, and hence, the studies were unable to ascertain the association between the risk factors and the observed infection clustering. Recently, a prevalence study by Morales and others found no clustering of porcine cysticercosis by household over what would be expected under natural environmental variation [9]. On the other hand, Lescano and others in Peru found an increasing clustering of porcine cysticercosis prevalence and incidence (based on antibody detection) towards households with tapeworm carriers [10]. The observed gaps in research for porcine cysticercosis as well as the conflicting findings from previous studies call for more research and emphasise the need for a context-specific situational analysis regarding the pattern of porcine cysticercosis to guide control efforts.

In Tanzania, a previous prevalence study in Mbulu district based on lingual examination of village pigs found clustering of porcine cysticercosis at village level [11]. Nevertheless, because the study was based on a less sensitive diagnostic test and the fact that it described clustering of the infection by a risk factor, it could not guide on specific geographical areas that needed more attention given limited resources to control the parasite. A Bayesian analysis of incidence data from a randomized field intervention trial based on health education found no clustering of the incidence rate ratio between the intervention and control villages [12]. Because of small sample sizes in many villages and exclusion of clustering by household, this study could not rule out clustering at village level. We found a need to identify priority geographical areas that needed more attention towards control of porcine cysticercosis in Mbulu district given limited resources. The present paper is based on secondary analysis of prevalence and incidence data collected in Mbulu district, northern Tanzania [12]. The study examined spatial clustering of porcine cysticercosis in Mbulu district, which was confirmed using Ripley's K functions and spatial scan statistics.

## Methods

### Ethics statement

This randomised field intervention trial is registered with the Australian New Zealand Clinical Trials Registry (ANZCTR), one of the WHO International Clinical Trials Registries. The registration number is ACTRN12609000190202. In Tanzania, the trial was approved by the Tanzania National Institute for Medical Research (NIMR) and Ministry of Health ethics review board, with reference number NIMR/HQ/R.8a/Vol.IX/88. The trial was also approved by Sokoine University of Agriculture (the principal researcher's institution) with reference number SUA/ADM/R.1/8, and the WHO/FAO Collaborating Center for Research and Training on Emerging and Other Parasitic Zoonoses, based at the Faculty of Life Sciences, University of Copenhagen, Denmark. Approval by these institutions was based on review of the research proposal. We obtained verbal consents from all study participants and their village authorities after the principal researcher had explained the purpose of and possible benefits from the study, as well as the freedom of the farmers to refuse participation. The verbal consents were recorded in a spreadsheet, which was finally approved by the institutional review board (the NIMR) before commencement of the field trial. We could not obtain written consents because of the high level of illiteracy (approximately 30%) in the study area and unwillingness to write by those who had some formal school education. Information from individual study participants were not disclosed to others. The use of the pigs in this research was based on the approval by the principal researcher's institution and the Mbulu District Livestock Department, which are responsible for animal care and use.

### Study area

Mbulu District is located in north-eastern Tanzania, between latitude 3.80° and 4.50° S, and between longitude 35.00° and 36.00° E. The altitude ranges from 1000–2400 m above mean sea level. The district contains areas having semi-arid and sub-humid climates that receive annual rainfall of <400 mm and >1200 mm, respectively. The long rainy season extends from March to mid-May and the short rainy period extends from November to December. Relative humidity ranges from 55 to 75% and mean annual temperature ranges from 15 to 24°C [13]. Currently, the District has 72 villages distributed in 16 wards, with an average of four villages per ward. The 2002 National census counted 237 882 people living in 38 729 households (average six persons per household). In 1997 the Mbulu District pig population was estimated at about 35 000, and crop and livestock production were by far the most important economic activities, employing >90% of the total labour force [13].

### Data

**Figure 1:**
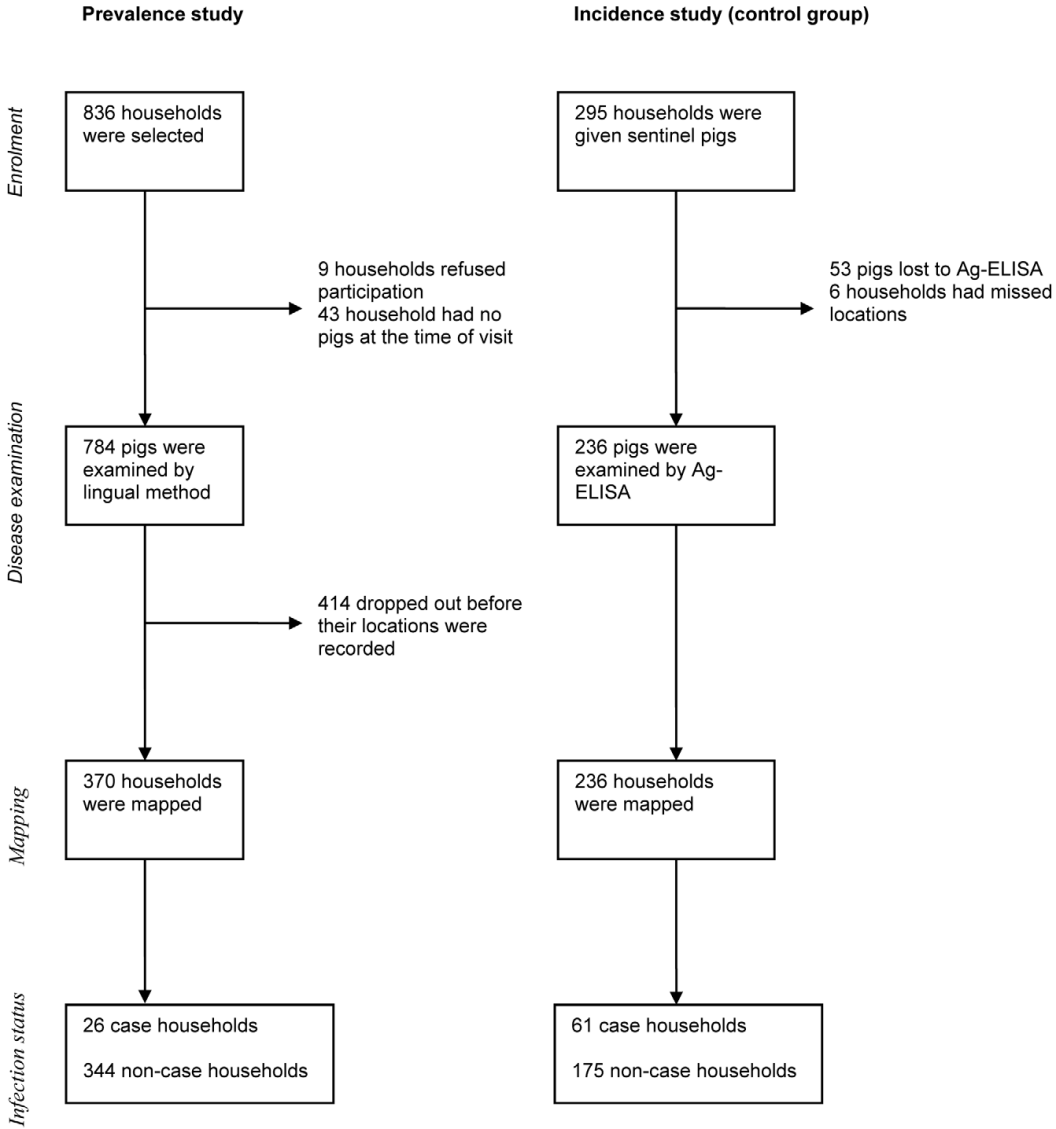
Flow of participants selected for analysis of spatial distribution of porcine cysticercosis prevalence in household pigs based on lingual examination, and of incidence in sentinel pigs based on Ag-ELISA. Mbulu district July 2002–April 2004.

This study is a secondary analysis of data collected during the baseline and follow-up periods of a randomized field trial that aimed at reducing the incidence rate of porcine cysticercosis through an educational programme. The baseline consisted of a cross-sectional study conducted between July 2002 and July 2003 to enable randomisation and evaluation of a health education intervention trial [12]. The baseline study was followed by a randomized controlled community trial conducted between July 2003 and April 2004. Sentinel piglets were provided to a random sample of pig-keeping households in the 42 villages and the effect of health education on the incidence rate of porcine cysticercosis and related transmission factors was evaluated after half of the villages had received a health education while the other half was left as control (received no intervention). The randomisation process was stratified by the baseline median prevalence of infection. More details of the methodology and some findings from this randomized trial have been provided elsewhere [12], [14], [15], [16]. The flow of participants during the randomised trial was described following the primary analysis of the data [12]. Figure 1 specifically shows the flow of participants selected for the present secondary analysis of the prevalence data and the incidence data based on lingual examination and antigen enzyme-linked immunosorbent assay (Ag-ELISA), respectively.

The village was the primary sampling unit. The choice of the number of villages to be included in the study was based on eligibility, whereby all eligible villages were included. The eligibility criteria for a village to participate in the study were that a village was keeping pigs, it had not been excessively studied for porcine cysticercosis, had ≥20 pig-keeping households, the village leaders agreed to participate, and the village was virtually independent from other villages. Based on the above criteria 42 out of 72 villages of the district qualified and they were enrolled in the study. Most of the villages excluded were administrative divisions of the studied villages [12]. In addition, two entire wards were excluded from the study because one (Yaeda Chini) was inhabited by a Bushmen society, which was not keeping animals, and the other (Mbulu Mjini) was occupied mostly by urban area of the district, where pig keeping was not popular.

Selection of pig-keeping households and pigs for the prevalence study was done randomly as described previously [12]. A total of 784 pig keeping households were included in the study, with the same number of pigs (one per household) examined for porcine cysticercosis prevalence. The eligibility criteria for a household to participate were presence of at least one pig 2–12 months old and willingness of the owner to participate the baseline and follow-up studies. Pigs aged 2 to 12 months were examined in the baseline study to match with a planned 12 months follow-up study [12]. Unfortunately, about 53% of participants dropped out after the baseline study. The reasons for the dropout was mainly poverty, though a few farmers were worried about an outbreak of African Swine Fever in a neighbouring district. Potential for selection bias was examined before proceeding to the follow-up study.

The randomized community-trial, which involved a follow-up of sentinel pigs to estimate incidence rates post-randomization, commenced immediately after the prevalence study. One out of the 14 studied wards was excluded in the present analysis because the randomization of the intervention by village assigned its village to the intervention group, hence not eligible for this analysis. Therefore, the total number of villages included in the control group for the incidence study was 21 located in 13 wards. Among the control group, a total of 295 households were provided with sentinel pigs (one per household) purchased in the district following screening with lingual examination. Blood samples were also collected and subsequently tested with Ag-ELISA. Briefly, approximately 3 ml of blood were collected from the jugular vein of each pig into a vacutainer tube. The samples were centrifuged in the same day and frozen at −21°C until their analysis, which was done after the end of field data collection. The Ag-ELISA was performed as described by Dorny and others [17]. The assay uses monoclonal antibodies to detect circulating *T. solium* antigens, an indication of presence of viable infection. All pigs included in the incidence study were free from infection based on both the lingual and Ag-ELISA. None of the pigs used in the prevalence study was used in the incidence study.

### Determination of baseline prevalence of porcine cysticercosis in household pigs

Upon arrival to a household, the selected pig was restrained in a standing position using a pig snare. A wooden rod was twisted gently between the lower and upper jaw, and by using a piece of cotton cloth, the tongue was gently pulled out and the under-surface visually examined for presence of cysticerci of *T. solium.* The examination was performed by the principal investigator (HAN) who had been trained in Veterinary Medicine and Veterinary Public Health.

### Determination of the incidence of porcine cysticercosis in sentinel pigs

Sentinel pigs aged 1–6 months (median 2 months) were reassessed once or twice following randomization to determine occurrence of cysticercosis using lingual examination and Ag-ELISA. For the pigs that completed the follow-up study, 32% were reassessed twice while 68% were reassessed once by Ag-ELISA. On the other hand, 44% and 56% were reassessed twice or once, respectively, by lingual examination.

### Collection of geographical data

In each study household, we recorded the location using a handheld geographical positioning system (GPS) receiver (Garmin, made in Taiwan) at an accuracy of 10 metres on average. All the geographical data were transferred to a field data sheet right in the field.

### Data analysis

Data were entered, coded, and cleaned in Microsoft Office Excel. Epi Info 3.5 was used to analyse the prevalence of porcine cysticercosis. We examined the potential for selection bias because of the participants dropout by performing three preliminary analyses to compare the distribution of 45 baseline variables between the dropouts and participants. In the first analysis, important baseline characteristics of the dropout participants were analysed to assess if there was any important difference between those who dropped out of the intervention and those who dropped out of the control groups. A total of 15 different variables were examined in this analysis. In the second analysis, the 15 variables were assessed to see whether there was any difference in the baseline proportions between the dropout and full participant households. In the final analysis, households that participated fully in the study were analysed to examine the distribution of the 15 baseline variables between the intervention and control group.

ArcView 3.2 was used to map the prevalence and incidence of porcine cysticercosis. We used ECESPA and SPATSTAT packages (available at http://cran.r-project.org/web/packages/) to compute Ripley's K functions in R statistical software to assess for general clustering of porcine cysticercosis. Bernoulli probability model was used in SatScan to identify local clusters of porcine cysticercosis prevalence at household level. Discrete Poisson model was used for the incidence data. In both cases, we used isotonic spatial scan statistic to take into account the heterogeneity of the study population. Ward specific relative risks of infections were estimated from the prevalence and incidence data using SatScan.

#### Mapping of the prevalence and incidence of porcine cysticercosis

All GPS points were projected to Universal Transverse Mercator (UTM) coordinate system, Spheroid Clarke1880 and later overlaid on the Mbulu district ward map using ArcView 3.2. The district ward map was extracted from the country ward map obtained from the International Livestock Research Institute (ILRI) website (http://www.ilri.org/gis/). We mapped the spatial point pattern for the prevalence and incidence studies separately. For the incidence study, results based on lingual examination and Ag-ELISA were presented in the same map, and only the control villages were included.

#### Spatial pattern analysis

Traditionally, the simplest theoretical model for a spatial point pattern is that of complete spatial randomness (CSR), in which the events are distributed independently according to a uniform probability distribution over a region [18]. However, in an epidemiological setting, such as that of this study, the hypothesis of CSR is implausible because of natural spatial variation in population density [19]. In this case, an observed spatial clustering of events could be due to natural background variation in the population from which events arise or due to a real biological effect. In such situations, “random labelling” hypothesis is considered the null hypothesis of no spatial clustering. In this case, the geographical distribution of subjects without the event under study (which we will call ‘controls’) describes the expected spatial pattern under natural environmental heterogeneity. The spatial point pattern for events of interest (e.g. cases) can be compared with that of controls. The random labelling hypothesis stipulates that the type of an event is independent of its location. Under the random labelling hypothesis, if *n*1 represents type 1 events of primary concern (cases) and *n*2 type 2 events that purport to represent environmental heterogeneity (controls) then, in the absence of clustering among the cases relative to the controls, if the two sets of events are pooled, the *n*1 case ‘labels’ would be attached at random to the combined set of events. The term “random labelling” originates from this concept. Ripley's K functions test consistency with or departure from spatial randomness.

Our spatial point pattern consisted of locations of households in which porcine cysticercosis events (present or absent) were recorded. Hence, the data represented a typical example of a “marked” point pattern. Our question was whether or not the observed porcine cysticercosis cases were clustered over and above the level that would be expected under natural environmental heterogeneity. We used Ripley's K functions to test our data for consistency with spatial randomness under random labelling. Under the random labelling hypothesis, it is expected that the K functions for cases (K_11_(d)) and for the controls (K_22_(d)) are identical throughout the distance (d) if there is no spatial dependence [20]. Thus, K_11_(d) =  K_22_(d) = K_12_(d) = K_21_(d) = K(d), and their differences would be zero [20]. In our study, K_11_(d) denotes the expected number of case households (labeled 1) within distance d of a randomly chosen case household. K_12_(d) denotes the expected number of control households (labeled 2) within distance d of a randomly chosen case household, and so on.

We computed K functions for the prevalence and incidence data separately. First we plotted the differences between the univariate K functions (K_11_(d)-K_22_(d)) for each dataset (prevalence, incidence) over distance ranging from 0–15 kilometers to test for clustering of porcine cysticercosis. Secondly, we plotted the differences between the univariate and the bivariate K functions (K_11_(d)-K_12_(d) and K_22_(d)-K_12_(d)) to evaluate the degree of segreggation of every individual pattern (cases, controls). The significance of departure from randomness was assessed by performing 1000-time simulations to establish upper and lower confidence limits. The choice of 1000 numbers of simulations was based on R software limitation. Nevertheless, for each model we repeated the analysis five times to increase the confidence of the interpretation. We based our significance test on simulation envelopes shown in the form of graphics rather than actual P values. For the incidence data, we analysed separately the results from Ag-ELISA and those from lingual examination. Some of the commands used in the computation of K functions are presented in Appendix S1. The choice of the distance range of 0–15 kilometers was based on recommendation by Dixon who suggested that for better estimation of the K functions, one should consider distances shorter than one-half the shortest dimension of the study area [21]. The approximate full distance of our study area in both axes was 76 kilometers. The model diagnostics for our data suggested the use of distance range of 0–14.7 km.

If there is no departure from randomness, any of the K function differences plotted would be be presented as a straight line passing through zero in the y axis parallel to the x axis. With clustering, the function would run above zero. The clustering would be considered statistically significant if the function was above the upper confidence limit. On the other hand if the function lies below zero, it indicates unlikely spatial clusering.

#### Analysis for local clustering of porcine cysticercosis

We used the Bernoulli probability model and discrete Poisson model in SatScan to scan for areas of high rates of porcine cysticercosis prevalence and incidence, respectively, at household level. For the incidence data, we used the total pig-months at risk for each pig to represent the population at risk for the household. We performed 9999 Monte Carlo replications to obtain precise estimates of P values and confidence limits. In both the prevalence and incidence data, we used isotonic spatial scan statistics to take into account the heterogeneity of the study population. We used a circular window in the study area with a maximum spatial cluster size specified at 50% of population at risk as recommended (22). With this specification, SatScan scanned for clusters of geographic size between zero and 50% of the population at risk. The population at risk for our prevalence study was the total sample size (n = 370) while for the incidence studies this was the total pig-months at risk for the Ag-ELISA (n = 927) and lingual examination (n = 1164). With the use of household as the unit of analysis, each household had a potential for becoming a centre of a cluster from which the radius of the cluster is measured. Statistical significance of a cluster was considered at P<0.05. We estimated ward specific relative risks of porcine cysticercosis by analysing the prevalence and incidence data at ward level using ward specific centroids. A ward specific relative risk is basically the risk of infection within the ward relative to that outside the ward (that is, in all other wards). A ward was considered to be at a low risk if its relative risk was ≤1 in both Ag-ELISA and lingual examination incidences. A ward was considered at a high risk if the relative risk in at least one of the two methods was ≥1. A ward with relative risk ≥1 in both the Ag-ELISA and lingual examination for incidence data was considered to be at a very high risk of infection. We excluded the prevalence data in this risk classification because of uncertainty as to whether or not a pig found infected during the prevalence study acquired the infection locally, and the fact that the prevalence study for the ward level analysis included 14 as opposed to 13 wards included in the incidence studies.

In SatScan, clusters are classified as “most likely clusters”, that is clusters that are least likely to be due to chance, and “secondary clusters” (other clusters detected in the data). P values for secondary clusters are calculated in the same way as for most likely clusters. A secondary cluster will only be considered important if it can reject the null hypothesis. In our study secondary clusters with P values >0.05 were ignored.

## Results

Following the analysis of 45 baseline variables for potential of selection bias as a result of considerable dropout after the prevalence study, the percentage of households using latrines was 8% lower (median difference) in the dropouts than in the participants (95% CI: −0.14, −0.03). The other 44 variables did not differ significantly (see Appendix S2). The one difference observed was most likely due to chance given that 45 variables were tested.

### Spatial distribution of porcine cysticercosis prevalence and incidence

**Figure 2:**
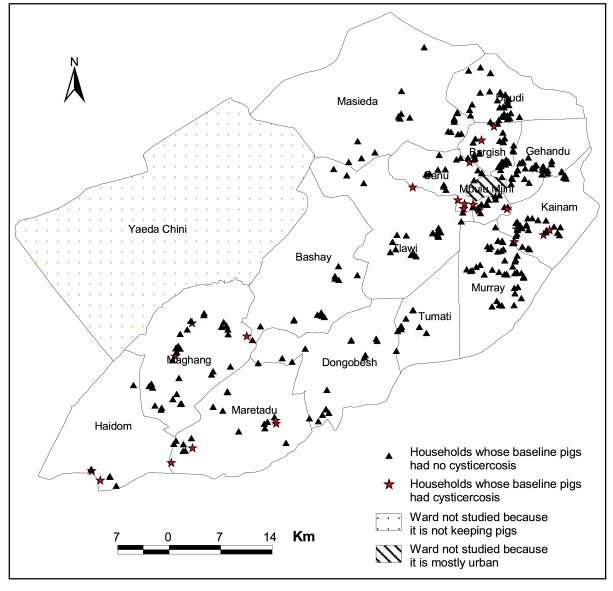
Spatial pattern (household level) of porcine cysticercosis prevalence based on lingual examination of household pigs in Mbulu district, northern Tanzania, 2002–2003.

The overall prevalence of porcine cysticercosis at the pig level was 7.3% (95% CI: 5.6, 9.4; n = 784) based on lingual examination. The incidence rate at the end of follow up of sentinel pigs was 69 (95% CI: 65, 72) per 100 pig-years and 25 (95% CI: 23, 28) per 100 pig-years, using Ag-ELISA and lingual examination, respectively [12]. The spatial point pattern of porcine cysticercosis prevalence in household pigs is presented in Figure 2. However, because of the dropout of some prevalence-study participants before their locations were recorded, the spatial map includes only 370 households.

**Figure 3:**
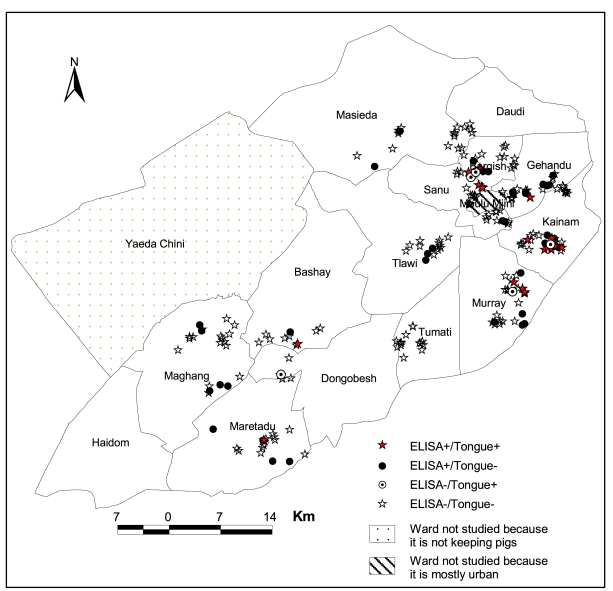
Spatial pattern (household level) of porcine cysticercosis incidence in sentinel pigs monitored for a median of 4 months (range: 2–9 months) using Ag-ELISA and lingual examination in Mbulu district, northern Tanzania, July 2003–April 2004.

The spatial distribution of porcine cysticercosis incidence in the sentinel pigs based on Ag-ELISA and lingual examination are shown in Figure 3. Locations of 6 of the 295 households included in the incidence study were not recorded. In addition, 53 and 48 households were lost to Ag-ELISA and lingual examination follow-up because their pigs died or got lost before reassessment. Therefore, the incidence study examined 236, 241, and 235 pigs by Ag-ELISA, lingual examination, and both tests, respectively. The median followed-up time was 4 months (range: 2–9 months).

### Spatial clustering of porcine cysticercosis in Mbulu district

#### General clustering

**Figure 4:**
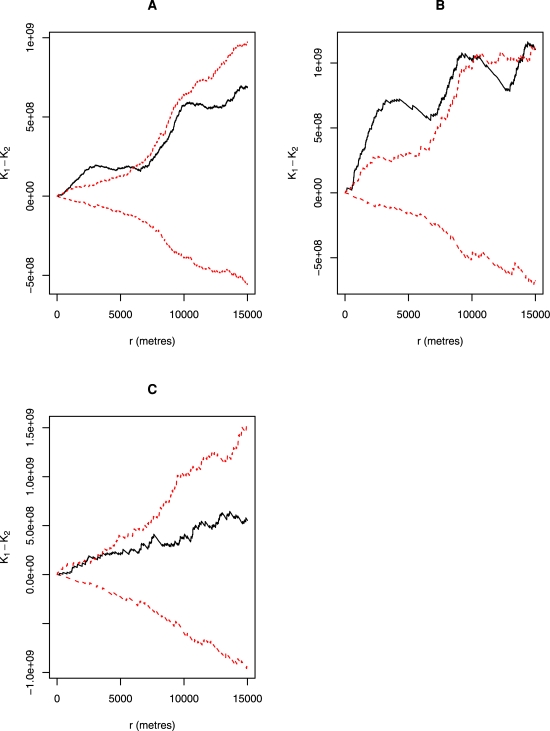
Differences between univariate K functions to test for spatial clustering of porcine cysticercosis, Mbulu district, Tanzania, 2003–2004. The difference between the univariate K functions is denoted here by K_1_-K_2_ while r represents the radius. (A) presents the results based on Ag-ELISA incidence while (B) and (C) present results based on lingual examination incidence and prevalence, respectively. Upper and lower 95% confidence limits for the K functions are indicated by dotted lines.

The Ripley's functions indicated a significant spatial clustering of porcine cysticercosis incidence over and above what would be expected due to natural environmental heterogeneity (Figure 4 (A) and (B)). While the Ag-ELISA incidence indicated significant clustering for all distances between 600 m and 5 km, the lingual examination method incidence indicated clustering from 650 m–6 km and between 7.5 and 10 km. On the other hand, the prevalence study did not indicate any significant clustering (Figure 4 (C)). With the use of simulation envelopes rather than actual P values to assess the significance of general clustering, we could not observe any difference between the results of the five repetitions done for each model. This is an indication of stability of the models, which resulted to apparently constant results between repetitions.

#### Segregation of case and control households

**Figure 5:**
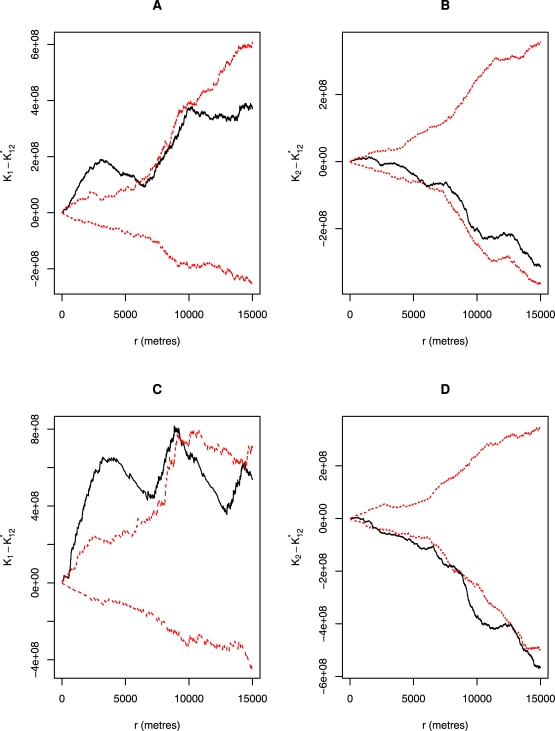
Differences between univariate and bivariate K functions to test for spatial segregation of each individual pattern, Mbulu district, Tanzania, 2003–2004. The difference between the univariate and bivariate K functions for case households is denoted here by K_1_–K*_12_. On the other hand, K_2_–K*_12_ denotes the difference for control households, and r is the radius. Figure 5 A and B show the results based on Ag-ELISA incidence while C and D present results based on lingual examination incidence. Upper and lower 95% confidence limits for the K functions are indicated by dotted lines.

It was evident based on Ag-ELISA and lingual examination results that households with incident cases of porcine cysticercosis tended to occur in proximity with other cysticercosis case households over the same distance ranges (Figure 5 (A) and (C)). On the other hand, control households did not show any trend towards aggregation, but rather regularity (Figure 5 (B) and (D).

#### Local clusters of porcine cysticercosis in Mbulu district

**Figure 6:**
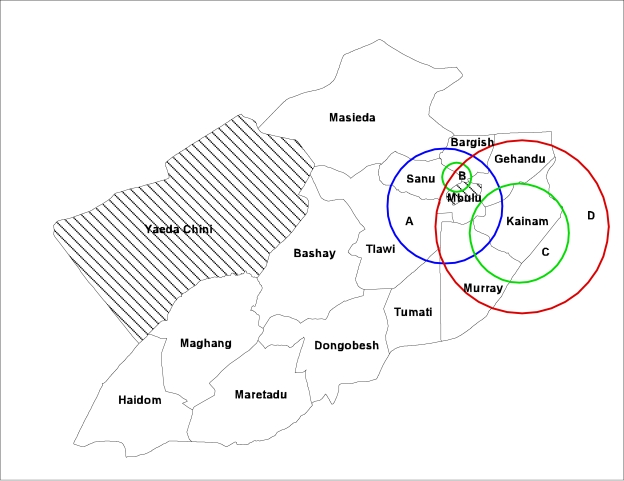
Local clusters of porcine cysticercosis in Mbulu Ditrict, Tanzania, 2003–2004. This figure describes local clusters of porcine cysticercosis identified by the prevalence study (circle A) and incidence studies based on lingual examination (circles B and C) and Ag-ELISA (circle D), as revealed by spatial scan statistical analyses at household level.

Spatial scan statistics revealed significant clustering of porcine cysticercosis in each study. There was one most likely cluster of porcine cysticercosis prevalence (Log likelihood ratio  = 12.984, P = 0.0036, radius  = 10.08 km). The Ag-ELISA data revealed one large most likely cluster of porcine cysticercosis incidence (Log likelihood ratio  = 13.2042, P = 0.0010, radius  = 15.18 km). On the other hand the lingual examination incidence data revealed one small most likely cluster (Log likelihood ratio  = 13.395, P = 0.0012, radius  = 2.54 km) and one significant secondary cluster (Log likelihood ratio  = 12.621, P = 0.0026, radius  = 8.68 km). There was overlap of the clusters, with the Ag-ELISA cluster containing most of the other clusters (Figure 6). There was a tendency for clustering nearby Mbulu district headquarter. Two clusters were centred at Sanu ward while two were cantered at Kainam ward. Table 1 shows ward specific relative risks of porcine cysticercosis incidences and prevalence. Out of the six wards included in the clusters, three (Sanu, Kainam, Murray) and one (Gehandu) were at a very high or high risk of infection, respectively, based on authors' classification. On the other hand, two wards (Bargish, Tlawi) were at a low risk of infection. The prevalence study also identified Sanu and Kainam as risky wards (RR = 4.2 and 2.0, respectively) while the remaining four wards were identified by this method as low risk wards.

**Table 1:** Ward specific relative risks (RRs) of porcine cysticercosis based on different studies.

Ward	Ag-ELISA incidence RR	Lingual exam incidence RR	Lingual exam prevalence RR	Authors' classification of relative risk*
Bargish	0.0	0.0	0.6	Low
Bashay	0.0	0.0	0.0	Low
Daudi	0.0	0.0	0.4	Low
Dongobesh	0.7	2.2	0.0	High
Gehandu	1.4	0.3	0.3	High
Kainam	2.2	4.2	2.0	Very high
Maghang	1.0	0.0	1.2	Low
Maretadu	1.3	0.6	3.0	High
Masieda	0.8	0.0	0.0	Low
Murray	1.6	1.3	0.0	Very high
Sanu	1.1	2.5	4.2	Very high
Tlawi	0.4	0.0	0.0	Low
Tumati	0.0	0.0	0.0	Low

*A ward was classified as very high risk if its relative risk by both Ag-ELISA and lingual examination incidence was >1 and high risk if only one of the incidence study gave a relative risk >1.

## Discussion

This study has for the first time described the spatial distribution of porcine cysticercosis in an endemic area of Africa. The study has also found that despite the low sensitivity of the lingual examination method for detecting porcine cysticercosis, it can be useful in identifying geographical areas where interventions should be directed. Nevertheless, the study suggests the need to use a combination of analytical procedures for effective identification of potential ‘hotspots’ of infections. One limitation of this study is lack of Ag-ELISA in the prevalence study, which limited assessment of the method in identifying clustering in cross-sectional studies and its comparison with the lingual examination prevalence results. In addition, the observed dropout of a large number of participants after the baseline study highlights on the need for careful recruitment of study participants intended for follow-up studies and analysis of potential for selection bias when losses to follow up are detected. This will enable the researcher to see whether or not results obtained from the remaining sample can be generalised to the target population.

The present study has established an overall significant clustering of porcine cysticercosis in Mbulu district of northern Tanzania, and identified significant local clusters of the infection. Lack of clustering of porcine cysticercosis for distances smaller than 600 m observed with Ripley's K functions could be due to omission of household clustering by the study design and the scattered nature of households in certain areas of the study district, such that only a few households would be found within the 600 m radius. The general significant clustering of porcine cysticercosis incidence revealed by the K functions was well supported by the presence of significant local clusters of the infections as determined by spatial scan statistics. The pattern of clustering of porcine cysticercosis incidence described by the K functions matched well with that found by spatial scan statistics, that is, the presence of one continous cluster by Ag-ELISA and two discrete clusters based on lingual examination method. One controversial finding in this study is the identification of a significant cluster of porcine cysticercosis prevalence by spatial scan statistics, but, failure of the K functions to recognise such clustering. Nevertheless, the fact that there is approximately 50% overlap of this cluster with that of Ag-ELISA incidence, there is a strong reason to believe that it is most likely cluster. Although possible low sensitivity of K functions to detect clustering of disease with low prevalence could be speculated, further studies are needed to ascertain this.

The use of various methods to identify clusters of porcine cysticercosis in this study has highlighted the potential strength of each method. The overlapping of clusters identified by the different methods strengthens the impression that the areas are ‘hotspots’. For example, the identification of Bargish and Tlawi wards as low risk wards by all three studies suggests that the wards are of low risk despite their inclusion in the clusters. On the other hand, the identification of Sanu, Kainam, Murray, and Gehandu as high risk wards by at least two of the methods, suggests that these wards are important areas of parasite transmission, calling for urgent attention. Although clusters from both the prevalence and incidence studies included the same wards, though at varying proportions, the cluster identified by the prevalence study was slightly differently located as compared to the clusters identified by the incidence studies. This could be due to the fact that the prevalence study included household pigs which we could not ascertain whether the observed infections were acquired locally given the dynamic nature of pigs in the area. Some of these pigs could have been introduced in the households while infected. For the incidence study, all pigs were serologically confirmed free from cysticercosis at the beginning of the study, and they were restricted from movement in terms of exchange until the end of the study. Therefore, infections that developed later on were most likely acquired at the household or at most around a small geographical area, where a pig could roam. The use of pig-months at risk as the background population at risk, which took into account the dynamic nature of the pig population, makes the incidence study results probably more relevant. The pig dynamicity may also explain the observed significant difference between the point prevalence and incidence rate determined by lingual examination despite the different sampling periods. The incidence study was able to record all cysticercosis incidents in the study population during the year, while the prevalence study captured the existing cases in the population at one time point. Some of cases could have been sold, moved, or died.

While the Ag-ELISA incidence identified one large cluster, the lingual examination incidence found two relatively small clusters, mostly contained within the Ag-ELISA cluster. This can be explained by the low sensitivity of the lingual examination method in detecting porcine cysticercosis, which could lead to the lingual examination being able to detect the cases later than the Ag-ELISA. Our results suggest that the lingual examination method is likely to be more sensitive where there is high infection pressure, for example, within the observed very high risk wards. It could also be possible that the actual cluster is irregular rather than circular as the SatScan could identify such a cluster as a series of small discrete clusters [22], in which case the lingual examination incidence cluster pattern would be more real. Unfortunately, there is no gold standard to ascertain the actual pattern of clustering in the study area.

Clustering of porcine cysticercosis in Mbulu district seemed to include certain wards, but not necessarily covering each ward entirely. However, for disease control purposes, we would recommend that any high risk ward included in the clusters be considered in totality because of shared administrative responsibilities and other common issues. In addition, despite the observed cluster boundaries (circle perimeters), it is not easy to identify them in the real world. It should also be noted that the observed Mbulu ward boundaries are apparently arbitrary, which could lead to misclassification of points if only visual impression is relied upon. This was the reason we also assessed the ward specific relative risks of infection using the actual administrative data and ward centroids. Nevertheless, spatial scan statistics could also identify high risk areas as low risk and vice versa. Thus, the need for multiple methods to identify clustering, the approach we used in this study.

The observed significant clustering of the incident cases is a possible indication of the clustering of parasite transmission risk factors. In May 2004 following the completion of the study, all study households received health education to reduce porcine cysticercosis. The health education consisting of training by a trained local livestock extension officer, a video show, and distribution of one booklet and leaflet to each participant was administered at village level. The present study recommends current situational analysis and use of combined interventions for ultimate elimination of the parasite.

Few studies have examined for possible clustering of *T. solium* infections in endemic areas. For example, in Peru, Garcia and others observed significant clustering of human cysticercosis seropositivity by the household whether or not the household had a tapeworm carrier [7]. Widdowson and others reported household clustering of porcine cysticercosis in Mexico [8]. However, both of these studies did not disentangle the clustering of the infections that could occur because of natural environmental heterogeneity. Recently, a prevalence study by Morales and others found no clustering of porcine cysticercosis by household over what would be expected under natural environmental variation [9]. A Bayesian analysis found no village-level clustering of the incidence rate ratio of porcine cysticercosis in the randomised trial where data for the present paper were derived. It is presumed that this could be due to the small number of pigs per village [12]. Lescano and others in Peru found an increasing clustering of porcine cysticercosis prevalence and incidence (based on antibody detection) towards households with tapeworm carriers [10]. Establishing the spatial patterns of porcine cysticercosis in an endemic situation is an important basis for implementing focused intervention given limited locations. Understanding the causal association as assessed by most of the previous studies is an important complementary to the findings of spatial pattern of the disease in order to efficiently implement appropriate interventions. Nevertheless, we emphasise the need to establish region-specific causes for clustering given conflicting findings elsewhere.

The overal prevalence of 7.3% observed in this study is lower than that of 17.4% reported previously in 21 villages of Mbulu district [11]. Several factors could explain the observed great difference in the prevalence between the two studies. The first and probably the most likely factor is the fact that the present study examined pigs from 2-12 months of age while the previous study included pigs from 2 months and above, about 84% of which were between 6 months and 5 years old [11]. The old pig population is likely to be mostly infected with cysticercosis because of the tendency of most smallholder pig farmers to keep infected pigs as breeding stocks due to lack of market. Secondly, the sampling of pig-keeping households in the previous study was haphazard, whereby any other household keeping pigs was visited after the previous household as opposed to the present study whereby the actual random sampling of the households was done. In addition, in the previous study all eligible pigs in a household were examined as opposed to the present study whereby only one pig per household was examined. Some of these factors could also account for the observed clustering of the infection by village in the previous study [11] but not in the consecutive incidence study in the area [12].

Several studies have shown that Ag-ELISA can detect two or more times as many cases of porcine cysticercosis as the lingual examination method [12]. Nevertheless, the lingual method has been reported to be highly specific (approximately 100%) [17]. Being easily available in the field and mostly accepted by the rural pig owners, the lingual examination method provides an avenue for monitoring porcine cysticercosis in an endemic situation. This was evident in the present study as indicated by the incidence study employing both the lingual examination method and Ag-ELISA. Both tests showed similar patterns of disease clustering, although with Ripley's K functions the lingual examination method suggested two peaks of clustering as opposed to one suggested by the Ag-ELISA. The good overlap of the clusters identified by spatial scan statistics between the two diagnostic methods further confirms the utility and potential limitation of the lingual examination. Future studies should examine for possible improvement of the lingual examination method. For example, in South Africa, Krecek and others [23] found that the use of additional light source to iluminate the oral cavity of the pig improved the accuracy of the test. This should be evaluated experimentally.

Note that the use of a combination of several methods to highlight on the spatial pattern of porcine cysticercosis in this study, though has increased our confidence on the results, might be too expensive to implement in endemic areas because of limited time and financial resources. Particularly, the use of incidence studies with sentinel pigs might have some practical and time limitations. This study recommends further studies to examine the ability of seroprevalence studies to identify hotspots of infection as a rapid way to guide implemention of control measures for *T. solium*. Once data on geographical locations and infection statuses have been obtained, subjecting the data to a variety of spatial and other analyses can be done to establish the spatial pattern of the infection or risk factors.

## Supporting Information

Alternative Language Abstract S1 Translation of the abstract into Spanish by David Carmena.
(0.05 MB DOC)

Alternative Language Abstract S2 Translation of the abstract into Chinese by Ying Zhang.
(0.07 MB RTF)

Alternative Language Abstract S3 Translation of the abstract into French by Pascal Nitiéma.
(0.13 MB RTF)

Appendix S1 Commands used in the computation of Ripley's K functions in R statistical software to test for clustering of porcine cysticercosis incidence based on Ag-ELISA in Mbulu district, northern Tanzania, 2003–2004.
(0.02 MB DOC)

Appendix S2 Comparison of the baseline characteristics between or within the households that dropped out of the study and those that participated to the end of the study in Mbulu District, northern Tanzania, 2002–2003.
(0.04 MB DOC)

Protocol S1 Trial protocol.
(0.15 MB DOC)
