# Supplementary material for: Spatial Clustering of Porcine Cysticercosis in Mbulu District, Northern Tanzania
Source: PLoS Negl Trop Dis. 2010 Apr 6;4(4):e652. doi: 10.1371/journal.pntd.0000652 (PMC2850315; doi:10.1371/journal.pntd.0000652)
Supplement: Alternative Language Abstract S1 — Translation of the abstract into Spanish by David Carmena. (0.05 MB DOC) [file pntd.0000652.s001.doc]

**Antecedentes**

La cisticercosis porcina es una enfermedad zoonótica causada por el cestodo Taenia solium que puede originar cuadros de enfermedad severos en humanos. El conocimiento previo tanto de la carga infectiva como del patrón de transmisión del parásito es esencial para el control efectivo de la enfermedad y la optimización de los recursos disponibles. El objetivo de este trabajo es el de establer la distribución espacial de la cisticercosis porcina en el distrito de Mbulu (norte de Tanzania) con objeto de servir de guía a posibles estrategias de control de la infección.

**Metodologia/Hallazgos principales**

Este estudio es un análisis secundario de los datos recogidos durante las fases inicial y de seguimiento de un ensayo comunitario aleatorio dirigido a reducir las tasas de incidencia de la cisticercosis porcina mediante la implantación de un programa educativo. Como situación de partida se seleccionaron aleatoriamente 784 núcleos familiares que mantuviesen cerdos, procedentes de 42 pueblos en 14 municipios. En cada núcleo familiar se efectuó el examen lingual de un cerdo escogido aleatoriamente (rango de edad: 2-12 meses, mediana: 8). El grupo control incluía 21 de los 42 pueblos estudiados, cuyos datos fueron usados en el estudio de incidencia. Un total de 295 cerdos sanos fueron suministrados a otros tantos núcleos familiares para ser usados como indicadores de la infección. Dichos animales fueron reanalizados una o dos veces en los 2-9 meses siguientes (mediana: 4) mediante examen ligual y ELISA para la detección de antígenos circulantes. La prevalencia de la cisticercosis porcina fue calculada usando la aplicación informática Epi Info 3.5. La distribución de la prevalencia y la incidencia de la enfermedad por núcleo familiar fue determinada usando la aplicación ArcView 3.2. Los agrupamientos globales y locales de la cisticercosis porcina fueron estimados calculando las funciones K y las estadísticas de Scan con las aplicaciones informáticas R y SatScan, respectivamente.

La prevalencia global de la cisticercosis porcina fue del 7.3% (95% CI: 5.6, 9.4; n=784). Las funciones K mostraron grupos globales de incidencia de la cisticercosis porcina significativos para todas las distacias comprendias entre 0.6 – 5 km desde un núcleo familiar aleatorio en base a los resultados de ELISA, y de 0.65 – 5 km y 7.5 – 10 km en base al examen lingual. El estudio de prevalencia no reveló ningún agrupamiento significativo por este método. Las estadistícas SatScan mostraron un grupo significativo de prevalencia de la cisticercosis porcina (P = 0.0036; n=370). Este análisis también reveló un gran grupo de incidencia de la cisticercosis porcina basado en los resultados de ELISA (P = 0.0010; n=236) y otros dos relativamente más pequeños basados en los exámenes linguales (P = 0.0012 and P = 0.0026; n=241). Todas las agrupaciones halladas presentaban una distribución espacial similar y afectaban a seis municipios, cuatro de los cuales fueron identificados como aéreas de elevado riesgo de cisticercosis porcina.

**Conclusión/Relevancia**

En este estudio se han identificado agrupamientos locales de cisticercosis porcina en el distrito de Mbulu, norte de Tanzania, donde los recursos destinados al control de T. solium son limitados. Se requieren más estudios para esclarecer las causas de estos agrupamientos e implantar intevenciones eficaces.
