# Supplementary material for: Spatial Clustering of Porcine Cysticercosis in Mbulu District, Northern Tanzania
Source: PLoS Negl Trop Dis. 2010 Apr 6;4(4):e652. doi: 10.1371/journal.pntd.0000652 (PMC2850315; doi:10.1371/journal.pntd.0000652)
Supplement: Alternative Language Abstract S2 — Translation of the abstract into Chinese by Ying Zhang. (0.07 MB RTF) [file pntd.0000652.s002.rtf]

北±坦¹桑£尼á亚Ç莫ª布¼鲁³区ø猪í囊Ò虫æ病¡的Ä空Õ间ä聚Û集¯性Ô
页³首×标ê题â: 坦¹桑£尼á亚Ç猪í囊Ò虫æ病¡的Ä聚Û集¯性Ô
研Ð究¿背³景°
猪í囊Ò虫æ病¡的Ä病¡原­体å,动¯物ï源´性Ô绦Ð虫æ----猪í肉â绦Ð虫æ可É在Ú人Ë类à引ý起ð严Ï重Ø的Ä疾²病¡症¢状´. 只»有Ð对Ô疾²病¡的Ä严Ï重Ø程Ì度È和Í特Ø征÷有Ð所ù了Ë解â, 合Ï理í地Ø分Ö配ä有Ð限Þ的Ä卫À生ú资Ê源´, 才Å能Ü对Ô这â种Ö寄Ä生ú虫æ病¡达ï到½有Ð效§控Ø制Æ. 本¾研Ð究¿的Ä目¿的Ä是Ç:找Ò出ö北±坦¹桑£尼á亚Ç莫ª布¼鲁³区ø猪í囊Ò虫æ病¡的Ä空Õ间ä分Ö布¼, 并¢以Ô此Ë指¸导¼控Ø制Æ该Ã病¡的Ä策ß略Ô.

研Ð究¿方½法¨和Í主÷要ª发¢现Ö
这â个ö研Ð究¿是Ç对Ô一»个ö社ç区ø随æ机ú干É预¤试Ô验é (目¿的Ä是Ç用Ã健¡康µ教Ì育ý来´降µ低Í猪í囊Ò虫æ病¡的Ä发¢病¡率Ê) 数ý据Ý的Ä二þ次Î分Ö析ö. 基ù线ß调÷查é及°随æ访Ã期Ú间ä所ù收Õ集¯的Ä数ý据Ý都¼包ü括¨在Ú内Ú。£共²有Ð十®四Ä个ö行Ð政þ区ø四Ä十®二þ个ö村å庄¯的Ä784户§养ø猪í人Ë家Ò被»随æ机ú选¡入ë基ù线ß调÷查é。£我Ò们Ç在Ú每¿户§都¼随æ机ú选¡取¡一»头·2到½12月Â大ó（¨平½均ù八Ë月Â）©的Ä本¾地Ø猪í作÷舌à检ì。£ 试Ô验é对Ô照Õ组é包ü括¨了Ë42个ö村å庄¯中Ð的Ä21个ö，¬这â些©村å庄¯就Í是Ç我Ò们Ç做ö这â次Î发¢病¡率Ê研Ð究¿的Ä对Ô象ó。£我Ò们Ç为ª295户§养ø猪í人Ë家Ò每¿户§提á供©了Ë一»头·哨Ú猪í，¬ 并¢在Ú2到½9个ö月Â的Ä时±间ä中Ð（¨平½均ù4个ö月Â）©用Ã舌à检ì和Í抗¹原­酶¸联ª免â疫ß吸ü附½测â定¨（¨ELISA）©进ø行Ð了Ë一»至Á二þ次Î囊Ò虫æ病¡发¢病¡的Ä再Ù评À价Û。£我Ò们Ç用ÃEpi Info 3.5计Æ算ã猪í囊Ò虫æ病¡的Ä患¼病¡率Ê,并¢用ÃArcView 3.2绘æ制Æ猪í囊Ò虫æ病¡患¼病¡和Í发¢病¡率Ê在Ú户§水®平½的Ä分Ö布¼图¼。£R软í件þ的ÄK函¯数ý被»用Ã于Ú评À价Û猪í囊Ò虫æ病¡的Ä普Õ遍é聚Û集¯性Ô。£SatScan 被»用Ã于Ú计Æ算ã扫¨描è数ý据Ý以Ô鉴ø别ð局Ö部¿感Ð染¾的Ä聚Û集¯性Ô。£
猪í囊Ò虫æ病¡的Ä总Ü患¼病¡率Ê是Ç7.3% (95%的Ä可É信Å区ø间ä: 5.6%, 9.4%; n=784)。£基ù于Ú抗¹原­酶¸联ª免â疫ß吸ü附½测â定¨的ÄK函¯数ý显Ô示¾: 在Ú任Î何Î随æ机ú选¡取¡的Ä一»户§人Ë家Ò的Ä600米×到½5千§米×范¶围§内Ú，¬猪í囊Ò虫æ病¡的Ä发¢病¡率Ê有Ð显Ô著ø聚Û集¯性Ô。£舌à检ì发¢现Ö的Ä聚Û集¯范¶围§为ª650米×至Á6千§米×，¬7.5千§米×至Á10千§米×。£同¬样ù的Ä方½法¨并¢未´发¢现Ö患¼病¡率Ê的Ä聚Û集¯性Ô。£Sat扫¨描è统³计Æ发¢现Ö猪í囊Ò虫æ病¡患¼病¡率Ê的Ä一»个ö显Ô著ø聚Û集¯区ø（¨P=0.0036;n=370）©。£同¬时±，¬根ù据Ý抗¹原­酶¸联ª免â疫ß吸ü附½测â定¨的Ä结á果û我Ò们Ç发¢现Ö了Ë一»个ö很Ü大ó的Ä猪í囊Ò虫æ病¡发¢病¡聚Û集¯区ø(P=0.0010;n=236),根ù据Ý舌à检ì的Ä结á果û发¢现Ö了Ë两½个ö相à对Ô较Ï小¡的Ä发¢病¡聚Û集¯区ø(P=0.0012 和ÍP=0.0026; n=241).这â些©聚Û集¯区ø都¼有Ð相à似Æ的Ä空Õ间ä位»置Ã,并¢且Ò都¼包ü括¨了Ë六ù个ö行Ð政þ区ø,其ä中Ð四Ä个ö行Ð政þ区ø被»划®为ª猪í囊Ò虫æ病¡高ß危£区ø.
结á论Û及°其ä显Ô著ø意â义å
这â个ö研Ð究¿在Ú北±坦¹桑£尼á亚Ç莫ª布¼鲁³区ø划®分Ö出ö局Ö部¿猪í囊Ò虫æ病¡聚Û集¯区ø, 这â样ù我Ò们Ç就Í可É以Ô将«有Ð限Þ的Ä控Ø制Æ猪í肉â绦Ð虫æ的Ä卫À生ú资Ê源´进ø行Ð有Ð效§分Ö配ä.进ø一»步½研Ð究¿造ì成É聚Û集¯性Ô的Ä原­因ò,可É以Ô帮ï助ú我Ò们Ç对Ô该Ã病¡进ø行Ð合Ï理í的Ä干É预¤.
