# Supplementary material for: Spatial Clustering of Porcine Cysticercosis in Mbulu District, Northern Tanzania
Source: PLoS Negl Trop Dis. 2010 Apr 6;4(4):e652. doi: 10.1371/journal.pntd.0000652 (PMC2850315; doi:10.1371/journal.pntd.0000652)
Supplement: Alternative Language Abstract S3 — Translation of the abstract into French by Pascal Nitiéma. (0.13 MB RTF) [file pntd.0000652.s003.rtf]

Agrégation spatiale de la cysticercose porcine dans le district de Mbulu, Tanzanie

Introduction
La cysticercose porcine est due au ver plat zoonotique Taenia solium qui peut causer des symptomes sévères chez l'homme. Afin d'allouer le peu de ressources disponibles dans un programme de contrôle, il est essentiel de bien comprendre le fardeau et la distribution de l'infection dans la population. L'objectif de cette étude est de décrire la distribution spatiale de la cysticercose porcine dans le district de Mbulu au nord de la Tanzanie afin de guider les décideurs dans la selection d'un programme de contrôle.

Méthodes / Résultats majeurs
Cette étude est une analyse secondaire des données de base et de suivi recueillies lors d'un essai de communauté randomisé qui avait pour but de réduire le taux d'incidence de la cysticercose porcine à l'aide d'un programme d'éducation. Lors de l'étude de base, 784 ménages élevant des porcs et résidant dans 42 villages et 14 quartiers ont été sélectionnés au hasard. La langue de tous les porcs âgés de 2 à 12 mois (médiane de 8 mois) a été examinée pour la présence de kystes. Un porc non infecté a été sélectionné au hasard dans chaque ménage. Les données sur le taux d'incidence de l'infection parmi les porcs élevés dans les 21 villages contrôles de l'essai randomisé ont été utilisées. Ces données proviennent de 295 porcs sentinelles donnés aux ménages participants et examinés pour la présence de l'infection à l'aide de l'examen de la langue et d'un test ELISA pour la détection des antigènes (AgELISA), une ou deux fois, soit de 2 à 9 mois (médiane de 4 mois), après la randomisation de l'intervention. La prévalence de la cysticercose porcine a été estimée à l'aide du logiciel Epi Info 3.5. La prévalence et le taux d'incidence de la cysticercose porcine ont été  apposés sur une carte des ménages à l'aide de Arc View 3.2. Les fonctions K ont été calculées à l'aide du logiciel R pour déterminer le degré d'agrégation de la cysticercose porcine. Les statistiques Sat Scan ont été utilisées pour identifier les agrégats d'infection locaux. 

La prévalence globale de la cysticercose porcine lors de l'étude de base était de 7.3% (95% IC: 5.6, 9.4; n=784). Les fonctions K ont démontré la présence d'agrégats du taux d'incidence, calculé à l'aide du test AgELISA, pour toute distance de 600 m à 5 km à partir d'un foyer infecté. Le diagnostic de l'infection par l'examen de la langue a permis d'identifier des agrégats de 650 m à 6 km et de 7.5 à 10 km. Aucun agrégat n'a été identifié par cette méthode de diagnostic lorsque les données de prévalence ont été utilisées. Cependant, les statistiques de Sat Scan ont permis d'identifier la présence d'un agrégat avec ces données de prévalence de cysticercose porcine (P = 0.0036; n=370).  Par ailleurs, cette méthode a permis d'identifier un important agrégat du taux d'incidence avec le test AgELISA (P = 0.0010; n=236) et deux plus petits agrégats avec l'examen de la langue (P = 0.0012 et P = 0.0026; n=241). Ces agrégats avaient la même position géographique et consistaient en six quartiers, lesquels ont été identifiés comme étant à haut risque pour la cysticercose porcine.  

Conclusion/Significance
Cette étude a permis d'identifier des agrégats spatiaux de cysticercose porcine dans le district de Mbulu, en Tanzanie du nord, où des ressources pourraient être investies pour le contrôle de T. solium.  D'autres études similaires devraient être menées dans l'objectif d'aider à mettre en place des interventions appropriées. 
