# Supplementary material for: Spatial Clustering of Porcine Cysticercosis in Mbulu District, Northern Tanzania
Source: PLoS Negl Trop Dis. 2010 Apr 6;4(4):e652. doi: 10.1371/journal.pntd.0000652 (PMC2850315; doi:10.1371/journal.pntd.0000652)
Supplement: Appendix S1 — Commands used in the computation of Ripley's K functions in R statistical software to test for clustering of porcine cysticercosis incidence based on Ag-ELISA in Mbulu district, northern Tanzania, 2003–2004. (0.02 MB DOC) [file pntd.0000652.s004.doc]

**Appendix S1**

**Commands used in the computation of Ripley’s K functions in R statistical software to test for clustering of porcine cysticercosis incidence based on Ag-ELISA in Mbulu district, northern Tanzania, 2003-2004**

> Sero <- read.delim("C:/Program files/R/R-2.7.2/Ngowi/Sero.txt", as.is=T)

> write.table(Sero, "Sero.dat") # Imports dataset and create a dataframe for use in R software

> SEROpoly <- as.points(Sero)

> bbox(SEROpoly) # Creates a bounding box of the study area

> SERO <- ppp(x=c(Sero$x), y=c(Sero$y), c(735240,794230), c(9534200,9602560),

+ unitname=c("metre","metres")) # Creates a point pattern for use in SPATSTAT package

> m1 <- (Sero$Ag-ELISA)

> m <- factor(m1, levels=0:1) # Identify marks of disease status (0=not infected, 1=infected)

> SEROmarked <- setmarks(SERO, m) # Marks the point pattern based on the disease status

> IncidenceSERO12 <- K1K2(SEROmarked, j="0", i="1", r=seq(0,15000),

+ nsim=1000, nrank=1, correction="isotropic") # Estimates the K functions with 1000 simulations

> plot(IncidenceSERO12$k1k2, lty=c(2, 1, 2), col=c(2, 1, 2), xlim=c(0, 15000),

+ main= "") # Plots the difference between the univariate K functions over the 0-15000 m distance

> plot(IncidenceSERO12$k1k12, lty=c(2, 1, 2), col=c(2, 1, 2), xlim=c(0, 15000),

+ main= "") # Plots the difference between the univariate and bivariate K functions for cases

> plot(IncidenceSERO12$k2k12, lty=c(2, 1, 2), col=c(2, 1, 2), xlim=c(0, 15000),

+ main= "") # Plots the difference between the univariate and bivariate K functions for controls.
