# Supplementary material for: Spatial Clustering of Porcine Cysticercosis in Mbulu District, Northern Tanzania
Source: PLoS Negl Trop Dis. 2010 Apr 6;4(4):e652. doi: 10.1371/journal.pntd.0000652 (PMC2850315; doi:10.1371/journal.pntd.0000652)
Supplement: Appendix S2 — Comparison of the baseline characteristics between or within the households that dropped out of the study and those that participated to the end of the study in Mbulu District, northern Tanzania, 2002–2003. (0.04 MB DOC) [file pntd.0000652.s005.doc]

Appendix S2 (a) Comparison of the baseline characteristics between or within the households that

dropped out of the study and those that participated to the end of the study in

Mbulu District, northern Tanzania, 2002-2003

| Factor | Within drop-outs (T- and C-group)^ұ^ | Between drop-outs and full participants | Within full participants  (T- and C-group) |
| --- | --- | --- | --- |
| Number of villages involved | 42 | 42 | 42 |
| Households involved (prop. Difference & 95% CI) | 0.03 (-0.04, 0.10) | Not-valid | -0.03 (-0.10, 0.04) |
| Knowledge on porcine cysticercosis (PCC) | -0.02 (-0.10, 0.07) | -0.070 (-0.13, -0.01) | -0.06 (-0.14, 0.03) |
| Knowledge on how a pig acquires PCC | 0.04 (-0.07, 0.15) | -0.01 (-0.09, 0.07) | 0.00 (-0.11, 0.12) |
| Knowledge on how to prevent PCC | 0.02 (-0.10, 0.13) | 0.02 (-0.07, 0.10) | -0.10 (-0.22, 0.01) |
| Knowledge on human taeniosis (HT) | -0.01 (-0.13, 0.12) | 0.07 (-0.02, 0.17) | -0.09 (-0.23, 0.05) |
| Knowledge on how a person acquires HT | -0.01, (-0.13, 0.11) | -0.10 (-0.20, -0.00) | -0.19 (-0.34, 0.04) |
| Knowledge on how to prevent HT | -0.07 (-0.18, 0.04) | -0.03 (-0.11, 0.06) | -0.09 (-0.21, 0.03) |
| Knowledge on PCC-HT relationship | 0.02 (-0.06, 0.10) | -0.04 (-0.11, 0.03) | -0.02 (-0.13, 0.09) |

^ұ^ T-group = Intervention group; C-group = Control group

Appendix S2 (b). Comparison of more baseline characteristics of households that dropped out of the

study and those that participated to the end of the study in Mbulu District,

northern Tanzania, 2002-2003

| Factor | Within drop-outs (T- and C-group) | Between drop-outs and full participants | Within full participants  (T- and C-group) |
| --- | --- | --- | --- |
| Household using latrine | 0.02 (-0.06, 0.11) | -0.08 (-0.14, -0.03)* | 0.06 (-0.02, 0.14) |
| Pigs that were found roaming | -0.01 (-0.10, 0.08) | 0.05 (-0.02, 0.11) | 0.03 (-0.06, 0.12) |
| Prevalence of porcine cysticercosis | 0.01 (-0.05, 0.06) | 0.01 (-0.03, 0.04) | -0.02 (-0.07, 0.03) |
| Pig gender ratio (% female) | 0.05 (-0.04, 0.15) | -0.00 (-0.07, 0.07) | -0.02 (-0.12, 0.08) |
| Pig age (mean difference) | 0.31 (-0.26, 0.89) | -0.06 (-0.47, 0.36) | -0.19 (-0.80, 0.41) |
| Who takes care of the pigs at home (mother) | 0.01 (-0.07, 0.10) | 0.06 (-0.00, 0.12) | -0.02 (-0.11, 0.07) |
