# Supplementary material for: Spatial Clustering of Porcine Cysticercosis in Mbulu District, Northern Tanzania
Source: PLoS Negl Trop Dis. 2010 Apr 6;4(4):e652. doi: 10.1371/journal.pntd.0000652 (PMC2850315; doi:10.1371/journal.pntd.0000652)
Supplement: Protocol S1 — Trial protocol. (0.15 MB DOC) [file pntd.0000652.s006.doc]

Title: EFFECTIVENESS OF HEALTH EDUCATION INTERVENTION FOR CONTROL OF PORCINE CYSTICERCOSIS IN MBULU DISTRICT, TANZANIA

PhD PROJECT PROPOSAL

PhD student: H.A. Ngowi^1^

Supervisors: A.A.Kassuku, M.R.S. Mlozi,^1^ J.E.D. Mlangwa,^1^ H. Carabin,^2^

A.L. Willingham^3^

**Institutional affiliations:** ^1^Sokoine University of Agriculture, Morogoro, Tanzania. ^2^ Department of Infectious Disease Epidemiology, Imperial College School of Medicine, St-Mary’s Campus, Norfolk Place, London, W2 1PG. ^3^Danish Centre for Experimental Parasitology, Royal Veterinary and Agricultural University, Denmark.

###### CONTENTS AT A GLANCE

- Summary
- Introduction
- Justification
- Objectives
- Materials and Methods
- Additional considerations
- Budget
- References

SUMMARY

Cysticercosis is a parasitic zoonosis caused by a tapeworm, *Taenia solium*, which infects both humans and pigs. *T. solium* causes serious diseases in humans and economic losses in endemic areas. Public health education has been reported to have a significant effect in reducing transmission of *T. solium* infections and it is likely to bring about long-term control of *T. solium* infections. Health education is suggested to be a priority even where other control strategies are intended. Since pigs are the primary intermediate hosts, they are obvious targets for the implementation of control measure. In addition, given the short life expectancy of the pig and the fact that it is difficulty to know when the infection occurs, the prevalence of porcine cysticercosis is a reliable indicator of active transmission zones. Effective health communication strategies require not only information on disease epidemiology and the characteristics of the technical intervention being promoted, but also social, cultural, behavioural, and economic data on the communities where the interventions will be implemented. In northern Tanzania, high prevalence of porcine cysticercosis was found following a study conducted in Mbulu district between 1998 and 1999. Lack of latrines, allowing pigs to roam, and lack of knowledge on the disease transmission were found to be factors likely contributing to the transmission of the disease in the area. Little is known on the economic losses occurring to farmers in Mbulu due to prevalence of porcine cysticercosis. Although the disease has been known by the farmers for many years, it is not known how the disease is perceived by the farmers. This information is necessary for evaluating the effectiveness of a control strategy in the area. The present study is intended to design and evaluate the effectiveness of a health education intervention for control of porcine cysticercosis in Mbulu district. The overall objective of the study is to reduce transmission of *T. solium* infections in humans and pigs in the area. The study will have three phases. Phase one of the study will be collection of anthropological data to aid in designing educational messages and study questionnaire relevant to the local context. Phase two will be designing and production of health educational materials. Phase three will be an assessment of the impact of the produced health educational materials in which a controlled randomised field trial will be conducted. The trial will have pre- and post-intervention examination of the study population in which both anthropological and epidemiological data will be collected for evaluation of the intervention. The community will participate in various activities during the research. The total duration of the study will be four years and three months effectively from July 2 000. The project will cost about US $ 93 739.97.

INTRODUCTION

Cysticercosis is a parasitic zoonosis caused by larvae of the tapeworm *Taenia solium*, which infect both humans and pigs. In humans the disease is called human cysticercosis whereas in pigs, it is called porcine cysticercosis. Human is the natural definitive host carrying the adult tapeworm in the small intestine. The adult worm causes a disease known as taeniosis that occurs when a person has eaten infected pork that may be raw or inadequately cooked. When the person ingests viable cysts from infected pork, the protoscolex evaginates in the intestinal tract and attaches to the mucosa of the small intestine, the proglottids develop from the neck of the protoscolex and mature as new ones are formed. Approximately three months after ingestion of the cyst, gravid proglottids start to appear in faeces, each containing about 60 000 eggs (Soulsby, 1982). Environmental contamination with infected human faeces is an important factor in sustaining the life cycle of *T. solium* (Schantz *et al.,* 1992). In the absence of treatment the adult worm may persist for up to 25 years in infected humans (Soulsby, 1982). This long-term holding of a person carrier of the tapeworm infection is of great epidemiological importance. It is known that invasive *T. solium* eggs may survive over six months in the external environment (Soulsby, 1982). The pig is the natural intermediate host harbouring the larval form of the parasite in the muscles and nervous tissues. Occasionally humans ingest the eggs of the parasite from faeces of infected humans through contaminated foods or water and develop human cysticercosis (Baily, 1996). When an intermediate host takes in eggs, onchospheres hatch in the small intestine and is activated under the influence of gastric and intestinal juices and penetrates the intestinal mucosa into blood and lymph vessels to be disseminated throughout the body. In pigs the onchospheres develop into larvae, *Cysticercus cellulosae* within 60 to 70 days (Miyazaki, 1991). Though the longevity of viable cysticerci is not known, the young age at which pigs are slaughtered means that the majority of cysts in pork would be viable.

The epidemiological cycle of *T. solium* occurs mostly in environments with low socio-economic or low hygiene levels, deficient sanitary facilities, or poor pig rearing practices where pigs can gain access to human faeces. Cysticercosis is most common in developing countries of Latin America, Asia, and Africa (Plancarte *et al.,* 1999). However, due to migration of people and tourism, human cysticercosis is now transmitted world-wide and it is considered as an emerging disease in the United States of America (Flisser *et al.,* 1998). Human cysticercosis has been reported in non pork-consuming communities such as the orthodox community in the USA (Schantz *et al.,* 1992). Studies on *T. solium* infections in Tanzania have largely been based on the infections in pigs, however, the possible medical implication of the parasite has been suggested by the presence of hospital records of human epilepsy and taeniosis in the studied areas (Ngowi, 1999; Boa, unpublished report). A study conducted at household level in 21 randomly selected villages in Mbulu district northern Tanzania, established an overall prevalence of 17.4% (range 3.2-46.7%) of porcine cysticercosis in 770 live pigs examined by lingual examination (Ngowi, 1999). This prevalence is higher than those reported by some similar studies in other highly endemic regions of the world. For example in Nigeria 5.5% of 1 300 pigs examined were found to be infected (Onah and Chiejina, 1995) while in Mexico, a prevalence of 4% was reported in 571 live pigs examined (Sarti *et al.,* 1992). Mbulu district was found to have a high potential for the spread of *T. solium* infections due to identified transmission factors. Out of 425 households observed, 21.6% did not have latrines. Of 198 respondents to interviews, 94.4% allowed their pigs to roam for food, and 41% of the respondents lacked knowledge on how a pig could acquire cysticercosis (Ngowi, 1999). Thus, public health education could contribute in reducing the risk of this severe disease. Several studies have found that neither age nor sex of the pig had a significant association with the prevalence of porcine cysticercosis (Sarti *et al.*, 1992; Onah and Chiejina, 1995; Sakai *et al.*, 1998; Ngowi, 1999). Studies in young pigs have indicated that infection rates are significantly higher in dry than in wet season (De-Aluja *et al.*, 1998; Ngowi, in process). In the study in Mbulu, this was linked to the fact that during dry season pigs are let free to look for food in the fields since at this time crops have been harvested. On the other hand during wet season pigs are housed or tethered to restrict them from destroying crops in the fields. Seasonal prevalence of porcine cysticercosis should be considered when targeting and evaluating control measures.

*T. solium* is of public health as well as economic importance in endemic areas. When the larvae of *T. solium* infect the central nervous tissues of human they can cause various neurological symptoms. Epileptic seizures have been found to be typical symptoms when the brain of human being is infected (Sarti *et al.,* 1992). This condition is referred to as neurocysticercosis and may be fatal if left untreated. Porcine cysticercosis is thought to cause serious economic losses in the pork industry due to costs related to condemnation of infected carcasses. However, little has been investigated on the actual economic losses occurring due to these condemnations and whether farmers perceive these losses in endemic areas. Porcine cysticercosis prevents small-scale farmers in Mbulu from marketing their pigs because pig traders reject pigs with lingual cysts. However, the monetary or bartering losses to the farmers associated with the condemnation of infected pigs in the area is not known. Knowing the economic losses caused by condemnation of infected pigs would be important for cost-benefit analysis of the control program and hence decisions on whether or not to implement the program. This information would also be useful in showing the farmers that it is profitable to change their current traditional pig management practices. Cost-benefit analysis is also important when guiding the health authorities in setting priorities. It is apparent that people in Mbulu have been aware of the presence of porcine cysticercosis for many years (Boa *et al.,* 1995; Nsengwa and Mbise, 1995; Ngowi, 1999). However, it is not known how the disease is perceived by the community. Studying people’s perception on the disease and its impact would help to know how much effort is required in the control of the disease. It is also the aim of the study to investigate if there are any social cultural, economic, and political factors that might cause barriers to community change.

Diagnosis of porcine cysticercosis in live pigs has been based on the examination of the under-surface of the tongue and by serology. Tongue examination has been found to be of a fair sensitivity and high specificity for field screening of porcine cysticercosis (Gonzalez *et al*., 1990). This method was found to detect up to 70% of truly infected pigs and it was 100% specific (Gonzalez *et al*., 1990). In Mbulu district (Boa *et al.*, 1995) and many other endemic areas (Sarti *et al*., 1992; Onah and Chiejina, 1995; Garcia-Noval *et al,* 1996) pig traders use the tongue examination method to reject owner’s pigs if they are found to harbour cysts under the tongue. Nevertheless, the predictive value negative of tongue examination is thought to be low since the method is less sensitive particularly in early or lightly infected pigs (Sciutto, *et al.*, 1998). Serology has been found to be relatively more sensitive compared to the tongue examination method (Gonzalez *et al*., 1990). Several serological methods have been developed elsewhere. However, most of these methods have limited application particularly in endemic areas due to passive transfer of maternal antibodies through colostrum, which have been found to persist beyond 8 months of pig’s age (Gonzalez *et al*., 1999). Therefore positive serology in endemic areas should be done with care when evaluating control strategies (Gonzalez *et al.*, 1994). Enzyme-linked immunosorbent assay (ELISA) method using purified antigen has been reported to be 100% sensitive and specific for human cysticercosis (Ito *et al.*, 1998) and it was reported to have similar efficacy in pigs. (Ito *et al*., 1999).

Different strategies have been advocated for the control of *T. solium* infections in endemic areas. These include treatment of infected humans or pigs, vaccination of pigs, general improved sanitation and industrialisation of pig rearing, and public health education (Plancarte *et al.,* 1999). Presently there is no vaccine available although there are promising progresses of finding a vaccine for porcine cysticercosis (Lightowlers, 1999). Despite the presence of effective drugs for both treatment of infected humans and pigs, strategies directed to the treatment of these hosts have limited applicability particularly in developing countries due to poor living conditions since the treated individuals are susceptible to re-infection (Lightowlers, 1999; Gonzalez *et al.,* 1999). Poor hygiene, economic constraints, and potential for re-introduction of the infections by migration of people are major problems that hinder the control strategies directed solely to treatment of infected hosts or general sanitation and industrialisation of pig rearing (Plancarte *et al.,* 1999). Studies carried out in Mexico for instance showed that there were no significant changes in porcine cysticercosis following treatment of human carriers of the adult tapeworm (Flisser *et al.,* 1998). Public health education is known to be valuable in reducing transmission of *T. solium* (Flisser *et al.,* 1998; Lightowlers, 1999)*.* This can be used alone or in combination with the other control strategies and in addition to controlling *T. solium* infections, it can reduce occurrence of other diseases related to poor hygiene such as cholera and typhoid fever. Public education has less economic demand, thus, can be implemented even in poor societies. Educational intervention is also important because it might bring permanent changes conducive to control and eventually eliminate the disease. Some educational interventions have shown significant reduction in the prevalence of porcine cysticercosis, significant changes in knowledge and some practices related to transmission of *T. solium* (Sarti *et al.,* 1997). A study in Mexico reported that after approximately one year post-intervention the prevalence of porcine cysticercosis decreased significantly (Sarti *et al.,* 1997). There is, however, lack of information on how public education can be effectively delivered to the target community. Various educational materials or communication channels such as radio programs, video, television, posters, books, pamphlets, and games can be used to educate farmers. Each medium has been found to have advantages and disadvantages, so using a number of methods to disseminate messages has been recommended to be more appropriate than relying on a single medium (Lloyd *et al.,* 1994). Effective health communication strategies require not only information on disease epidemiology and the characteristics of the technical intervention being promoted, but also social, cultural, behavioural, and economic data on the communities where the interventions will be implemented (Keilbach *et al.,* 1989; Lloyd *et al.,* 1994; Sarti *et al.,* 1997). Due to differences in social cultural, economic, and political settings in different areas of the world, there is a need to determine the effectiveness and sustainability of a control program in an endemic situation. The present study is intended to evaluate the effectiveness of a health education intervention in reducing transmission of *T. solium* infections in Mbulu district, Tanzania.

JUSTIFICATION

Pig rearing provides subsistence in many families in Mbulu district in northern Tanzania and is a source of animal protein in some other densely populated towns and cities of Tanzania such as Arusha, Moshi, and Dar es Salaam where most pigs from Mbulu are sold for slaughter and consumption. Before 1980s pigs from Mbulu were also exported to Kenya for commerce. However, porcine cysticercosis cut off the market in Kenya in early 1980s after the exported pigs were found to be infected (Mbulu district veterinary officer, verbal communication). Improving the health of the rural pigs in Mbulu would therefore improve the economic states of the rural farmers, increase food security in the district, reduce incidence of human taeniosis and cysticercosis, return the export opportunity, and improve the country’s economy.

Cysticercosis prevents small-scale farmers in Mbulu from marketing their pigs because pig traders reject pigs with lingual cysts. The monetary or bartering losses to the farmers associated with the condemnation of infected pigs in Tanzania is not known. Knowing the economic losses caused by condemnation of infected pigs would be important for benefit-cost analysis of the control program and hence a decision on whether or not to implement the control program. This information is also necessary to show farmers that it is profitable to change their current traditional pig rearing practices. Benefit-cost analysis is also important in guiding the government authorities when setting priorities. Mbulu district is found to have a high potential for the spread of *T. solium* infections due to identified risk factors such as lack of latrines, allowing pigs to roam, and poor knowledge on the mode of transmission of the parasite (Ngowi, 1999). Thus, public health education could contribute to reduce the risk of this severe disease. It is apparent that people in Mbulu have been aware of the presence of porcine cysticercosis for many years (Ngowi, 1999). However, it is not known how the disease is perceived by the community. Studying people’s perception on the disease is necessary in order to know how much effort is required in the control of the disease.

OBJECTIVES

**General objective:**

To reduce transmission of *Taenia solium* infections in humans and pigs in Mbulu district, Tanzania.

**Specific objectives:**

(i) To assess human perceptions and practices related to *T. solium* infections in the area.

(ii) To describe the epidemiology of porcine cysticercosis and assess its economic impact in Mbulu district.

(iii) To evaluate the effectiveness of produced health educational materials in improving human perceptions and practices related to the transmission of the disease.

(iv) To evaluate the effectiveness of the health educational materials in reducing the prevalence of porcine cysticercosis.

MATERIALS AND METHODS

##### Study area

Mbulu District is located in north-eastern Tanzania, between latitude 3.80^o^-4.50^o^S and longitude 35.00^o^-36.00^o^E. The altitude varies from 1000-2400 m above sea level. The area has a semi-arid and sub-humid climate and receives an annual rainfall of less than 400 mm and over 1200 mm respectively. The long rainy season extends from about March to Mid-May and the short rainy period extends from November to December. Relative humidity ranges from 55%-75% and mean annual temperature ranges from 15^o^C-24^o^C. The District has 59 villages and the 1997 District census showed that it had a population of 200 000 people and 32 883 pigs. Crop and livestock production is by far the most important economic activity, employing over 90% of the total District labour force. Most of the inhabitants are Iraqw tribe that occupies most of the highland areas and practices mixed farming. Pigs are raised in most households as a source of income and animal protein for the household. Most of the pigs are let free to graze particularly after harvesting. When there are crops in the fields most pigs are tethered at the backyard and are fed on cut grass, kitchen leftovers, pumpkins and maize grains. Few farmers house their pigs. Slaughtering of pigs takes place at home although few of the pigs are slaughtered at a nearby open slaughter slab. Some home slaughtered pigs are inspected by a meat inspector while some are not. Besides pig farming, villagers also raise other livestock including cattle, sheep, goats, and chicken. Some villagers keep dogs, cats, as well as donkeys.

##### Study design

This study will have three phases. Phase one will be a pilot study in which anthropological data will be collected to aid in designing appropriate educational messages and the study questionnaire. Phase two will be designing and production of health educational materials. Phase three will be an assessment of the impact of the produced health educational materials.

## Activities

## Phase

Collection of anthropological data to aid in designing relevant educational messages and study questionnaire

1

# 2

Designing and production of health educational materials

3

Dissemination and evaluation of health educational materials

Controlled randomized trial

Pre-intervention examination

Intervention

Post-intervention examination

-Perceptions

-Practices

-Prevalence of PCC

-Perceptions

-Practices

-Prevalence of PCC

Phase one - Pilot study:

A pilot study will be carried out in Mbulu district for the main purpose of collecting anthropological data to aid in designing appropriate health educational messages and study questionnaire. The pilot study will investigate the acceptance of the study by the community by making formal contacts with the local government authorities from the district to the village level to introduced the purpose of the study and seek permission to carry out the study. A meeting with study participants selected from each of the selected villages will be organised to ask for informed consent. Using key informants and participatory data collection methods, the pilot study will investigate community perceptions and practices related to *T. solium* transmission. The informants will be selected to represent farmers in different age groups and gender, health workers, pig traders, and veterinarians. Focus group discussions will be conducted consisting of the above categories. Each focus group will consist of six people and several group discussions will be conducted until sufficient information is attained. In-depth interviews will involve one person in each of the categories mentioned above. Therefore, a total of 7 individuals will be included in the in-depth interviews. The study will assess the local perception of the people about the disease, transmission, and control. The study will investigate whether the disease is perceived of any importance by assessing if there is any control effort that has been taken against the disease. The study will also investigate if there is any kind of action taken by the meat inspectors in case of infected pork and by health workers in case of taeniosis and epileptic patients. The community will be asked to give their recommendations for behavioural change and possible barriers. The village included in these anthropological studies will be excluded from the main study. The pilot study will also be used for informal testing of the questionnaire intended for the main study. A village extension agent will be selected in each village under study and trained by the researcher to be the health educator for the village participants. Updated census of pig farmers will be collected from the district to aid in deciding how many villages to be enrolled in the study.

##### Phase two - Designing and production of health educational materials:

The designing and production of educational materials will be done with the community participation. A video program, brochures, posters, and overhead transparencies will be in accordance with the local understanding. The general theme of the education will be to address the community on the life cycle of *T. solium*, transmission factors, impacts, and preventive and control measures. Emphasis will be put on constructing good latrines using locally available resources, hygienic use of latrines, washing hands before and after meal, building pig houses using locally available materials and feeding pigs indoor using locally available food stuffs. Other useful messages suggested by the community will be included.

Phase three - Dissemination and evaluation of health educational materials:

##### This will be a controlled randomised intervention trial with pre- and post-intervention examination of the study population. The pre-intervention study will be concerned with assessing the prevalence of porcine cysticercosis and its economic impact to the farmer. The study will also assess human perceptions and practices related to the transmission of *T. solium* infections in the area. Following the pre-intervention study, an intervention will be done in which participants in half of the selected villages will receive the health education while half of the villages will act as control. The village will be the unit of intervention allocation. Allocation of the villages to either intervention or control group will be done on a random manner or matched if randomisation will seem to favour communication between groups. Post-intervention study will be conducted to re-assess human perceptions and practices related to the disease, and the prevalence of porcine cysticercosis. The independent variables are therefore the prevalence of porcine cysticercosis, human perceptions and practices related to the disease, while the dependent variable will be the health education.

**Study population**

Sample size calculation:

Although the village will be the unit of intervention allocation, the main analysis will be based on individuals since from previous study there was little variation in the prevalence of porcine cysticercosis in different villages (coefficient of variation was about 0.1). Therefore the sample size has been calculated using the formula,

n = [(z_1_ + z_2_)^2^ 2P (1-P)]/(p_1_ – p_2_)^2^.

In the formula, n is number of pigs required in each group, z_1_ is the z score for the specified confidence level, and z_2_ is the z score for the specified power. p_1_ and p_2_ are the prevalence of the disease in the intervention and control group respectively, and p_1_ and p_2_ P is the average of p_1_ and p_2_ (Smith and Morrow, 1996).

The sample size is calculated based on the prevalence of porcine cysticercosis as the main outcome measure. The prevalence of porcine cysticercosis in the area was estimated to be 14.64% adjusted for various confounders (Ngowi, 1999). We would like the present study to have 80% power to detect a significant difference with 95% confidence if the intervention will reduce the prevalence of porcine cysticercosis by 30%. The number of villages required in each group is therefore calculated to be:

n = [(1.96 + 0.84) ^2^ 2 x 0.1244 (1-0.1244)]/(0.1464-0.1023)^2^

= 878.20.

Approximately 900 pigs will be examined in each of the two groups. A total of 1800 pigs will be examined before intervention and 1800 after intervention.

Selection of villages and study participants:

The number of villages to be included in the study will depend on the number of pig farmers available in the villages. This information will be obtained in the pilot study. All pig farmers in the selected villages will be enrolled in the study.

##### Data collection methods and experimental procedure

##### Pre-intervention examination of the study population:

A structured questionnaire will be used to collect demographic data of all the study participants. In-depth interviews of five farmers in each village will be done to study human perceptions of the disease. Structured observations will be done in all selected households to study practices related to the transmission of the disease such as pig rearing practices, use and status of latrine, boiling of water for drinking, washing hands before eating and after using a latrine, and eating of infected pig meat.

All pigs in the household will be examined by the researcher using lingual palpation and blood samples collected for antigen ELISA.

# Allocation of villages to intervention:

Two groups will be compared whereby one group will receive the health education while the other group will act as a control. The villages will be grouped according to their similarities in their prevalence of porcine cysticercosis obtained during pre-intervention study and randomised within groups to either control or intervention.

Intervention:

Participants in the control group will follow their usual hygiene and pig management practices. For the intervention group, the educator in each village will organise and conduct two training sessions one- month interval. After the first session the educator will distribute pamphlets to the participants for further reference. The day after the session, the participants in the intervention group will watch a video program. The researcher will participate in the sessions as an audience, therefore the interventions in the selected villages will be planned such that the researcher will participate in all the villages.

# Post-intervention examination of the study population:

The researcher will reassess perceptions and practices immediately after the intervention and at the end of the study. All pigs born after the intervention will be examined. Similar methods of data collection used in the pre-intervention study will be used in this study. However, the last questionnaire interview will include a question designed to investigate if the participants in the control group have received any information regarding the health education from the intervention group in order to take care for possible confounder during data analysis. The research will also investigate if there has been any change in hygiene policy at any time during the study.

**Statistical analysis**

Due to the fact that pigs will be clustered in households that are also clustered in villages and climates, a hierarchical logistic regression model will be used to analyse the data in order to take care of different levels of clustering and possible interactions. The effectiveness of intervention in improving human perceptions and practices will be computed by comparing average scores in the two groups adjusted for baseline values. Change in the prevalence of porcine cysticercosis will be computed and adjusted for important confounders such as age, sex of the pig, and other important household characteristics that might affect the prevalence of the disease. Seasonal prevalence of porcine cysticercosis will be taken into considerations when evaluating the intervention.

# In the calculation of economic losses by the farmer it shall be assumed total condemnation of the infected pig. This is the regulation of the government and it is practised by most people in Mbulu district. The prevalence of porcine cysticercosis in all one-year old pigs in the control group will be used to estimate the economic losses by the farmer assuming that the pig is sold at one year of age. The average price of a pig will be estimated using the existing price.

The economic loss will be calculated as the loss in net returns due the disease prevalence + costs incurred due to condemnation of infected pig carcasses.

**Activity time plan**

| **Activity** | **Year 1** | **Year 2** | **Year 3** | **Year 4+** |
| --- | --- | --- | --- | --- |
| Develop research proposal | X |  |  |  |
| Attend Research Methodology course | X |  |  |  |
| Phase 1 (collect anthropological data) |  | X |  |  |
| Attend Applied Epidemiology course |  | X |  |  |
| Phase 2 (produce ed. Materials) |  | X |  |  |
| Phase 3 (evaluate ed. Materials) |  | X | X | X |
| Preliminary data analysis |  |  |  | X |
| Community feedback and evaluation |  |  |  | X |
| Final data analysis and publication |  |  |  | X |

ADDITIONAL CONSIDERATIONS

##### Ethical considerations

The researcher will ask for informed consent from the study participants before she can carry out the study. Confidentiality of the study participants will be adhered to. Ethical clearance will be requested from the National Institute for Medical Research ethical clearance committee in Tanzania. Research permission will also be requested from the researcher’s home institution (Sokoine University of Agriculture, Tanzania). After the end of the study the control group participants will receive the intervention. If the study participant or the family member is ill in our presence, she/he will be carried by the project vehicle to the health centre. If the animal of the study participant is sick the farmer will be advised to seek for the veterinary advice. If the intervention will be found effective it will be considered for the district.

**Study limitations**

Due to the short period available for the data collection (about two years), it might not be possible to observe significant reduction in the disease prevalence. However, improvement in perceptions and practices related to the transmission of the disease will be used as intermediate outcomes to indicate reduction in the transmission of the disease.

Maternal antibodies due against porcine cysticercosis have been reported to persist beyond nine months of pig’s life. This may limit the use of ELISA or immunoblot tests that have been reported to respond on these antibodies. This is because we would like to detect actual cases (and not false positives due to maternal antibodies) and to be able to disentangle cases hat occured before and those that occurred after the intervention. Most studies on antigen ELISA for porcine cysticercosis are still under trial and some have reported that antigens are detected up to 200 days post-infection and tend to disappear afterwards probably due to formation of immune complexes.

We are not sure that we will get enough pigs for post-intervention study since we don’t know how prolific are the pigs in the study area. From the previous study, one farmer in Mbulu had an average of 1.8 pigs. Our aim is to examine a different population of pigs in the post-intervention from the pre-intervention study. This is because in this particular disease we cannot know the onset of the infection, otherwise we could include the pre-intervention population that had not been infected in the post-intervention study.

Due to the fact that the intervention is information based, leaking of information from intervention to the control group might be a problem. However care will be taken to have a clear geographical separation of the groups. In addition, participants in the control group will be assured well in advance that they will receive the intervention after the study in order to minimise chances of them seeking information from the intervention group. The researcher will investigate if there is any leakage of information from the intervention to the control group, or if there is any change in hygiene policy during the study period in order to take care of these during data analysis. [In this point I am still thinking if we can use blinding which is known to reduce bias]

There might be barriers to community change due to the fact that some change in practices such as housing pigs may require additional resources to cater for extra labour and food. The pilot study will investigate the possible barriers and take them into considerations when evaluating the effectiveness of the program.

Currently there is an outbreak of African swine fever in the capital city of Tanzania that has resulted in restriction of pig movements and slaughtering of all infected pigs. It is not known how far this will spread and for how long it will last. This might affect the pig population in Tanzania and might have some impact in the present study.

##### Sharing of facilities with other projects

This project is among several projects under DANIDA ENRECA Livestock Helminths Research Project in Tanzania. The present project will be able to share facilities already available in the project such as vehicles, refrigerators, and desktop computers. The project will also be able to use institutional facilities such as ELISA reader, incubator, and laboratory space.

**Dissemination of research results**

Findings from this research will be disseminated to various relevant parts. The community feedback will be through organising workshops and seminar with the study population. Written reports will be given to the district and village authorities. A thesis will be produced and a copy will be available in the institutional library. Scientific papers will be written and published in relevant journals. Oral or poster presentations will also be given in relevant scientific conferences and workshops.

BUDGET

| **Requirement** | Cost | |
| --- | --- | --- |
|  | TAS | **US $^*^** |
| *Equipments* |  |  |
| A video system (deck + screen) | 600 000 | 750 |
| An electric generator to drive the video in the villages | 300 000 | 375 |
| Tape recorder & 10 cassettes for questionnaire recording | 220 000 | 275 |
| 1 pig snare for restraining the pig during examination | 100 000 | 125 |
| **Sub-total** | **1 220 000** | **1 525** |
| *Materials* |  |  |
| 1videotape production | 19 000 000 | 23 750 |
| 1 000 brochures | 700 000 | 250 |
| 1 000 forms for farmers to keep records | 100 000 | 125 |
| Travels associated with media production | 300 000 | 375 |
| 100 m of a cotton material @ 1000/= for holding pig’s tongue during examination | 100 000 | 125 |
| **Sub-total** | **20 200 000** | **25 250** |
| *Allowances and accommodation* |  |  |
| 15 months of data collection in Mbulu:   - - - Per diem for 3 (researcher, driver, technician) people @ 14 000/day     - Anthropologist to assist on anthropological data collection (allowance to be negotiated)     - Accommodation: 8 000/person/day | 18 900 000  10 800 000 | 23 625  13 500 |
| **Sub-total** | **29 700 000** | **37 125** |
| *Local travel costs for the 15 months* |  |  |
| [Morogoro – Arusha-Mbulu (800 km) + return] x 15 months x 700/= / 8 km  Within Mbulu 50 km/day (estimated)  Vehicle maintenance (10% fuel cost) | 2 100 000  3 543 750  564 375 | 2 625  4 429.69  7 054.69 |
| **Sub-total** | **6 208 125** | **7 760.16** |
| *Overseas travel costs* |  |  |
| Research methodology course (4.5 months)  -return air ticket to Denmark  -subsistence allowance 480 000/= per month  Thesis write up (9 months)  - return air ticket to Denmark  -subsistence allowance 480 000/= per month | 1 563 200  2 160 000  1 563 200  4 320 000 | 1 954  2 700  1 954  5 400 |
| **Sub-total** | **9 606 400** | **12 008** |
| *Other costs* |  |  |
| Fuel for running the generator | 20 000 | 25 |
| Stationery and costs for communication (e-mail, fax, Tel.) | 500 000 | 625 |
| First aid kits | 20 000 | 25 |
| Incentives | 500 000 | 625 |
| Thesis production | 200 000 | 250 |
| **Sub-total** | **1 240 000** | **1 550** |
| Contingency to cater for inflations and emergencies (10% of total budged) | 6 817 452.5 | 8 521.82 |
| **Sub-total** | **6 817 452.5** | **8 521.82** |
| **Grand total** | **74 991 977.5** | **93 739.97** |

REFERENCES

Baily, G.G. (1996) Cysticercosis. In: *Manson’s Tropical Diseases, 20^th^ edition. (Edited by Gordon C. Cook)* WB Saunders Company Ltd, London, Philadelphia, Toronto, Sydney, Tokyo. pp 1779.

Boa, M.E., Bogh, H.O., Kassuku, A.A. and Nansen, P. (1995) The prevalence of *Taenia solium* metacestodes in pigs in northern Tanzania. *Journal of Helminthology* 69, 113-117.

De-Aluja, A.S., Martinez, M.J.J. and Villalobos, A.N. (1998) *Taenia solium* cysticercosis in young pigs: age at first infection and histological characteristics. *Veterinary Parasitology* 76, 1-2, 71-79.

Flisser, A., Sarti, E., and Schantz, P. (1998) New strategy for controlling human cysticercosis. *Proceedings for the 9^th^ International Congress of Parasitology* 179-184.

Garcia-Noval, J., Allan, J., Fletes, C., Moreno, E., Mata, F. D., Torres-Alvarez, R. and Mencos, F. (1996) Epidemiology of *Taenia solium* taeniasis and cysticercosis in two rural Guatemalan communities. *American Journal of Tropical Medicine and Hygiene* 55, 3, 282-289.

Gonzalez, A. E., Cama, V., Gilman, R. H., Tsang, V. C. W., Pilcher, J. B., Chavera, A., Castro, M., Montenegro, T., Verastegui, M., Miranda, E. and Bazalar, H. (1990) Prevalence and comparison of serologic assays, necropsy and tongue examination for the diagnosis of porcine cysticercosis in Peru. *American Journal of Tropical Medicine and Hygiene* 43, 2, 194-199.

Gonzalez, A.E, Falcon, N., Gavidia, C., Garcia, H.H., Tsang, V.C.W., Bernal, T., Romero, M. and Gilman, R.H. (1999) Treatment of porcine cysticercosis with oxfendazole: a dose-response trial. *The Veterinary Record* 420-422.

Gonzalez, A.E., Gilman, R., Garcia, H.H., McDonald, J., Kacena, K., Tsang, V.C., Pilcher, J.B., Suarez, F., Gavida, C. and Miranda, E. (1994) Use of sentinel pigs to monitor environmental *Taenia solium* contamination. The Cysticercosis Working Group in Peru (CWG). *American Journal of Tropical Medicine and Hygiene* 51, 6, 847-850.

Ito, A., Plancarte, A., Ma, L., Kong, Y., Cho, S.Y., Liu, Y.H., Kamhawi, S., Lightowlers, M.W. and Schantz, P.M. (1998) Novel antigens for neurocysticercosis: simple method for preparation and evaluation for serodiagnosis. *American Journal of Tropical Medicine and Hygiene* 59, 2, 291-294.

Ito, A., Plancarte, A., Nakao, M., Nakaya, K., Ikejima, T., Piao, Z.X., Kanazawa, T. and Margono, S.S. (1999) ELISA and immunoblot using purified glycoproteins for serodiagnosis of cysticercosis in pigs naturally infected with *Taenia solium*. *Journal of Helminthology* 73, 4, 363-365.

Keilbach, N.M., Aluja, A.S. and Sarti-Guiterrez, E. (1989) A program to control Taeniasis-cysticercosis (*T. solium*): Experiences in a Mexican village. *Acta Leidensia* 57, 2, 181-189.

Lightowlers, M.W. (1999) Eradication of *Taenia solium* cysticercosis: a role for vaccination of pigs. *International Journal of Parasitology* 29, 811-817.

Lloyd, L. S., Winch, P., Ortega-Canto, J. and Kendal, C. (1994) The design of a community-based health education intervention for the control of *Aedes aegyti. American Journal of Tropical Medicine and Hygiene* 60, 4, 401-411.

Miyazaki, I. (1991) *Helminthic zoonoses.* Shukosha Printing, Fukuoka, Japan. pp 494.

Ngowi, H. A. (1999) Endoparasites of zoonotic importance in pigs in Mbulu district, Tanzania. *Masters thesis*. pp 69.

Nsengwa, G.R.M. and Mbise, A. N. (1995) Porcine cysticercosis in Tanzania: preliminary findings. *Bulletin of Animal Health and Production in Africa* 43, 2, 161-162.

Onah, D. N. and Chiejina, S. N. (1995) *Taenia solium* cysticercosis and human taeniasis in the Nsukka area of Enugu State, Nigeria. *Annals of Tropical Medicine and Parasitology* 89, 4, 399-407.

Plancarte, A., Flisser, A., Gauci, C.G. and Lightowlers, M.W. (1999) Vaccination against *Taenia solium* cysticercosis in pigs using native and recombinant oncosphere antigens. *International Journal of Parasitology* 29, 643-647.

Sakai, H., Sone, M., Castro, D.M., Nonaka, N., Quan, D., Canales, M., Ljungstrom, I. and Sanchez, A.L. (1998) Seroprevalence of *Taenia solium* cysticercosis in pigs in a rural community of Honduras. *Veterinary Parasitology* 78, 3, 233-238.

Sarti, E., Flisser, A., Schantz, P.M., Gleizer, M., Loya, M., Plancarte, A., Avila, G., Allan, J., Craig, P., Bronfman, M. and Wijeyaratne, P. (1997) Development and evaluation of health education intervention against *Taenia solium* in a rural community in Mexico. *American Journal of Tropical Medicine and Hygiene* 56, 2, 127-132.

Sarti, E., Schantz, P.M., Plancarte, A., Wilson, M., Gutierrez, I.O., Lopez, A.S., Roberts, J. and Flisser, A. (1992) Prevalence and risk factors for *Taenia solium* taeniasis and cysticercosis in humans and pigs in a village in Morelos, Mexico. *American Journal of Tropical Medicine and Hygiene* 46, 6, 677-685.

Schantz, P.M., Moore, A.C., Munoz, J.L., Hartman, B.J., Schaefer, J.A., Aron, A.M., Persaud, D., Sarti, E., Wilson, M. and Flisser, A. (1992) Neurocysticercosis in an Orthodox Jewish community in New York City. *New England Journal of Medicine* 327, 692-695.

Sciuto, E., Martinez, J.J., Villalobos, N.M., Hernandez, M., Jose, M.V., Beltran, C., Rodarte, F., Flores, I., Bodadilla, J.R., Fragoso, G., Parkhouse, M.E., Harrison, L.J. and De-Aluja, A.S. (1998) Limitations of current diagnostic procedures for the diagnosis of *Taenia solium* cysticercosis in rural pigs. *Veterinary Parasitology* 79, 4, 299-313.

Smith, P.G. and Morrow, R.H. (1996) *Field Trials of Health Interventions in Developing Countries: A toolbox.* MacMillan Education Ltd, London and Basingstoke. pp 362.

Soulsby, E.J.L. (1982) *Helminths, Arthropods and Protozoa of Domesticated animals*. Baillière Tindall, London, 7th edition. pp 809.
